# Cs_3_Bi_2_Br_9_/g-C_3_N_4_ Direct Z-Scheme Heterojunction for Enhanced Photocatalytic Reduction of CO_2_ to CO

**DOI:** 10.1021/acs.chemmater.3c01635

**Published:** 2023-10-16

**Authors:** Yasmine Baghdadi, Filipp Temerov, Junyi Cui, Matyas Daboczi, Eduardo Rattner, Michael Segundo Sena, Ioanna Itskou, Salvador Eslava

**Affiliations:** † Department of Chemical Engineering and Centre for Processable Electronics, Imperial College London, London SW7 2AZ, United Kingdom; ‡ Nano and molecular system (NANOMO) research unit, University of Oulu, Oulu 90570, Finland; § Department of Graduation in Chemical Engineering, Universidade Federal do Rio Grande do Norte/UFRN, 59.078-970 Rio Grande do Norte, Brazil; ∥ Barrer Centre, Department of Chemical Engineering, Imperial College London, London SW7 2AZ, United Kingdom

## Abstract

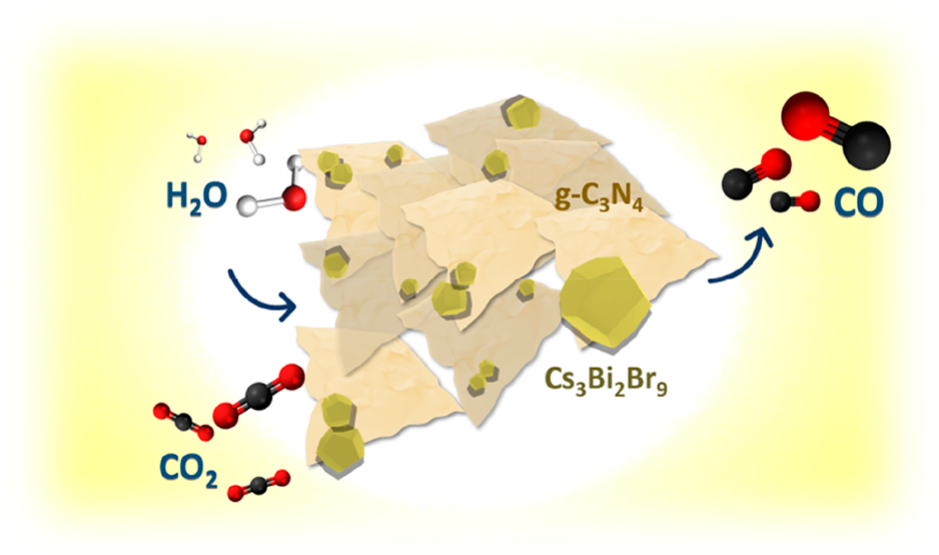

Lead-free halide perovskite derivative Cs_3_Bi_2_Br_9_ has recently been found to possess optoelectronic properties suitable for photocatalytic CO_2_ reduction reactions to CO. However, further work needs to be performed to boost charge separation for improving the overall efficiency of the photocatalyst. This report demonstrates the synthesis of a hybrid inorganic/organic heterojunction between Cs_3_Bi_2_Br_9_ and g-C_3_N_4_ at different ratios, achieved by growing Cs_3_Bi_2_Br_9_ crystals on the surface of g-C_3_N_4_ using a straightforward antisolvent crystallization method. The synthesized powders showed enhanced gas-phase photocatalytic CO_2_ reduction in the absence of hole scavengers of 14.22 (±1.24) μmol CO g^–1^ h^–1^ with 40 wt % Cs_3_Bi_2_Br_9_ compared with 1.89 (±0.72) and 5.58 (±0.14) μmol CO g^–1^ h^–1^ for pure g-C_3_N_4_ and Cs_3_Bi_2_Br_9_, respectively. Photoelectrochemical measurements also showed enhanced photocurrent in the 40 wt % Cs_3_Bi_2_Br_9_ composite, demonstrating enhanced charge separation. In addition, stability tests demonstrated structural stability upon the formation of a heterojunction, even after 15 h of illumination. Band structure alignment and selective metal deposition studies indicated the formation of a direct Z-scheme heterojunction between the two semiconductors, which boosted charge separation. These findings support the potential of hybrid organic/inorganic g-C_3_N_4_/Cs_3_Bi_2_Br_9_ Z-scheme photocatalyst for enhanced CO_2_ photocatalytic activity and improved stability.

## Introduction

To tackle global warming, researchers have been working on developing different techniques to process CO_2_ after its emission through capture, storage, or reduction into useful carbon products. Photocatalytic reduction of CO_2_ to solar fuels entails the use of a semiconductor capable of absorbing light and generating electron/hole pairs that participate in different surface redox reactions. For a CO_2_ reduction reaction (CO_2_RR) to take place, the chosen semiconductor is required to be stable under different reaction conditions, nontoxic, and cost-effective. In addition, the semiconductor should possess band energy levels that encompass the redox potentials for the target reactions. Different semiconductors such as TiO_2_, ZnO, SiC, and WO_3_ have been explored throughout the years for photocatalytic CO_2_RR under a variety of conditions.^1^ Recently, halide perovskites are seen as promising candidates for photocatalytic CO_2_ reduction due to their light harvesting capability, efficient charge generation,^2^ long carrier diffusion lengths,^3^ and a band structure aligned with CO_2_ redox potentials.^4^ Among the different atomic combinations that form the ABX_3_ perovskite structure (A and B as organic or inorganic cations and X as an anionic halide), halide perovskites with lead (Pb) in the B-site have received much attention in the photocatalytic field due to their wide utilization in solar cells.^5,6^

Currently, the most heavily researched lead halide perovskites are CsPbBr_3_ or CH_3_NH_3_PbI_3_ owing to their abundant elements.^7^ However, perovskites with Pb^2+^ cations in their structure pose the risk of releasing the toxic element to the environment, since photocatalysts are not encapsulated like solar cells.^8,9^ To address this challenge, researchers have been testing perovskite-inspired materials that substitute lead with a combination of cations that preserve charge neutrality. As a result, a wide range of halide double perovskites (A_2_B^+^B^3+^X_6_ and A_2_B^4+^X_6_) and halide perovskite derivatives are being studied as a substitution.^10^ A derivative of the structure A_3_B_2_X_9_ can be synthesized using ternary cations [bismuth (Bi) or antimony (Sb)] such as Cs_3_Sb_2_I_9_, Rb_3_Sb_2_I_9_, or MA_3_Bi_2_Br_9_ (MA: methylammonium). Studies have shown that the perovskite derivative Cs_3_Bi_2_Br_9_ exhibits a two-dimensional topology consisting of corrugated layers of perovskite-like corner-sharing octahedra that results in an indirect band gap of approximately 2.59 eV.^11,12^ As a result of its absorption in the visible range, Cs_3_Bi_2_Br_9_ has shown photocatalytic activity to CO production from CO_2_ under simulated sunlight irradiation.

A promising approach to improve charge separation and reduce recombination in bismuth-based halide perovskites is to couple it with a second semiconductor with a slightly staggered band edge alignment.^13^ Once electron/hole pairs are generated individually in each material, electrons in the semiconductor with a higher conduction band (CB) edge will move to the CB of the second semiconductor, while the holes will move from the valence band (VB) of the latter to higher energy. This typical type-II heterojunction is known to improve charge separation and boost the overall photocatalytic efficiency.^14^ Alternatively, a staggered band edge alignment can also allow electrons in the CB of one semiconductor to combine with the holes in the second. Electrons accumulate on the surface of the semiconductor with a higher CB and the spatial charge separation further increases creating a direct Z-scheme configuration.^15^

Graphitic carbon nitrides (g-C_3_N_4_) are two-dimensional organic polymers formed mainly of s-triazine and tri-s-triazine repeat units. Studies show that g-C_3_N_4_ is a semiconductor with a typical band gap around 2.7 eV enabling it to absorb light with wavelengths below 475 nm.^16^ In addition to its suitable CB and VB edge positions, g-C_3_N_4_ can be synthesized using inexpensive and abundant precursors such as melamine or urea by a simple calcination process with a microstructure that can be easily tailored.^16^

While possessing an alignment in band energetics and complementary optoelectronic properties, research on Cs_3_Bi_2_Br_9_/g-C_3_N_4_ composites has been focused on H_2_ evolution and oxidation reactions.^17−19^ To date, little research has explored photocatalytic CO_2_ reduction in the gas-phase without the use of hole scavengers. In this work, we successfully synthesized a direct Z-scheme heterojunction between Cs_3_Bi_2_Br_9_ and bulk g-C_3_N_4_ using a straightforward antisolvent crystallization method. Each semiconductor was synthesized and characterized before optimizing the ratio between the two for a better performing heterojunction. It was observed that using 40 wt % Cs_3_Bi_2_Br_9_ with g-C_3_N_4_ led to the highest CO production of 14.22 (±1.24) μmol CO g^–1^ h^–1^ under simulated sunlight demonstrating a 2.5- and 7.5-fold improvement in activity in comparison to pure Cs_3_Bi_2_Br_9_ and g-C_3_N_4_, respectively. Photoelectrochemical measurements also confirmed the enhancement in charge separation by displaying higher photocurrents for the 40 wt % Cs_3_Bi_2_Br_9_. The hybrid organic/inorganic composite demonstrated improvement in stability with prolonged light exposure. The optoelectronic characterization of the composite indicates the formation of a Z-scheme heterostructure. Further selective metal-deposition analysis was used to verify that the heterostructure possessed a direct Z-scheme behavior with reduction occurring on the surface of Cs_3_Bi_2_Br_9_.

## Experimental Details

### Materials

CsBr (99%, Sigma-Aldrich), BiBr_3_ (99%, Sigma-Aldrich), melamine (99%, Alfa Aesar), dimethyl sulfoxide (≥99.9%, Sigma-Aldrich), dihydrogen hexachloroplatinate (IV) hexahydrate (Thermoscientific), and anhydrous 2-propanol (99.5%, Sigma-Aldrich) used in this work were used as received. Fluorine-doped tin oxide coated (FTO) glass and tetrabutylammonium hexafluorophosphate (TBAPF_6_) were purchased from Sigma-Aldrich. Extra dry (99.9+%) acetonitrile was purchased from Acros Organics and stored in a glovebox with controlled nitrogen atmosphere (<0.5 ppm of H_2_O, <0.5 ppm of O_2_).

### Synthesis of Cs_3_Bi_2_Br_9_ Crystals

Cs_3_Bi_2_Br_9_ was synthesized using an antisolvent crystallization process. In detail, 1.2 mmol of CsBr and 0.8 mmol of BiBr_3_ were added to 10 mL of dimethyl sulfoxide. The solution was stirred at room temperature for 2 h to ensure the complete dissolution of the bromide salts. The solution was then added swiftly into a round-bottom flask containing 500 mL of isopropanol and stirred vigorously for 1 min. The resulting bright yellow suspension was washed and centrifuged 3 times with anhydrous isopropanol at 10000 rpm to remove excess dimethyl sulfoxide. The obtained powder was dried overnight at 40 °C in a vacuum oven and stored in a nitrogen atmosphere in a glovebox.

### Synthesis of g-C_3_N_4_

Bulk g-C_3_N_4_ was obtained through the direct calcination of melamine. A total of 5 g of melamine were added to a 50 mL alumina crucible covered with a lid and heated at 10 °C min^–1^ up to at 550 °C in air. This temperature was maintained for 4 h and then left to cool naturally to room temperature. The coarse g-C_3_N_4_ was gently grinded into a finer powder with a mortar and pestle.

### Synthesis of Cs_3_Bi_2_Br_9_/g-C_3_N_4_ Composites

To prepare the Cs_3_Bi_2_Br_9_/g-C_3_N_4_ composite, 250 mg of the obtained bulk g-C_3_N_4_ was dispersed in 500 mL of isopropanol. The mixture was sonicated for 60 min to obtain a homogeneous dispersion of g-C_3_N_4_ in the antisolvent. The solution of precursors in dimethyl sulfoxide was then swiftly added to the g-C_3_N_4_ dispersion, and the mixture was stirred vigorously for 1 min. To modify the ratio of Cs_3_Bi_2_Br_9_ to g-C_3_N_4_, the amount of precursor was modified so that the nominal loading of Cs_3_Bi_2_Br_9_ varied between 10, 20, 40, 60, and 80 wt %. A physical mixture between the two materials was also obtained by mixing pure g-C_3_N_4_ and pure Cs_3_Bi_2_Br_9_.

### Characterization

Powder X-ray diffraction (XRD) was carried out using an Xpert Pro PANalytical diffractometer operated at 40 kV voltage and 20 mA current using Cu Kα (l = 0.15418 nm) radiation in the 2θ range 5–75°. Peak deconvolution was performed on OriginPro 2022b based on a Gaussian model with details shown in Table S1. The analysis of the diffractogram was conducted on MDI Jade 6. UV–vis diffuse reflectance spectroscopy (DRS) of the powder samples was conducted using a Shimadzu UV-3000 with an integrated sphere and barium sulfate (BaSO_4_) as a reference. The material band gap (*E*_*g*_) was calculated by extrapolation of the onset linear region of the curve using the Tauc plot of [*F*(*R*)*hν*]^1/*n*^ versus *hν* where *F*(*R*) represents the Kubelka–Munk function calculated using reflectance (*R*) data such that *F*(*R*) = (1 – *R*)^2^(2*R*)^−1^, *hν* is the light energy, and *n* is a constant equivalent to 0.5 for direct *E*_*g*_ and 2 for indirect *E*_*g*_ calculations. The composition of the prepared samples was obtained by CHN elemental analysis using a Thermo Fisher Scientific Flash 2000 analyzer. X-ray photoelectron spectrophotometry (XPS) was performed by using a Thermo Fisher K-Alpha+ with a monochromated Al Kα X-ray source to understand the elemental composition of the samples. In the same XPS machine, valence-band XPS and work function (⌀) measurements were carried out using gold as a reference. All binding energies were corrected with respect to adventitious carbon at 284.8 eV and all XPS data was processed using Avantage software. Scanning electron microscopy (SEM) was conducted using a Zeiss Auriga Cross Beam with a secondary electron detector. The powder samples were dispersed on silicon wafers and coated with 15 nm chromium using a Q150T Quorum pumped coater to improve surface conductivity. Transmission electron microscopy (TEM) was performed using a JEOL JEM-2100Plis microscope. Photoluminescence (PL) spectra of the powders were measured between quartz substrates by a photoluminescence spectrometer (FLS1000, Edinburgh Instruments). Excitation at 380 nm with a bandwidth of 10 nm (generated by a 450 W ozone-free continuous xenon arc lamp), a long-pass filter (395 nm cutoff wavelength), emission bandwidth of 5 nm, dwell time of 0.1 s, and spectral resolution of 1 nm was used. Each PL spectrum was individually normalized by its weight (pure semiconductors) or the weight of the g-C_3_N_4_ component in the composite. Fourier transform infrared (FT-IR) spectroscopy was performed using an Agilent Technologies Cary 630 spectrometer in the attenuated total reflection mode (ATR). Finally, Raman spectroscopy was conducted on a Bruker Senterra II spectrometer using a 785 nm laser source with a power of 25 mW. To test the mode of charge separation between the two semiconductors, photocatalytic deposition of platinum was performed using H_2_PtCl_6_·(H_2_O)_6_ in anhydrous isopropanol, and its location was mapped using SEM and energy dispersive X-ray (EDX) spectroscopy. A dispersion of 1 mg mL^–1^ of the photocatalyst in anhydrous isopropanol was prepared and illuminated with a 300 W Xe source from LOT-Quantum Design equipped with an AM1.5G filter adjusted to 1 sun (100 mW cm^–2^) intensity. After 1 h, the sample was collected and washed with anhydrous isopropanol and mapped by SEM and EDX. Electron paramagnetic resonance (EPR) was conducted in the solid state using a Bruker X-Band continuous-way Elexsys E500 system at 100 kHz frequency and 1 G amplitude. The powder samples were illuminated with a 365 nm monochromatic light source for 30 min before EPR measurement.

### Photocatalytic performance

Photocatalytic CO_2_ experiments on the as-prepared photocatalysts (PCs) were conducted in a 20 mL gastight stainless steel photoreactor with a quartz window. A 1 mg mL^–1^ dispersion of the desired sample in anhydrous isopropanol was drop cast on a 32 mm Cytiva Whatman quartz filter and dried on a hot plate at 80 °C for 30 min prior to performing the reaction. This was to ensure the use of a thin and homogeneous layer of the PC for the test. The filter was placed in the center of the photoreactor, and 40 μL of distilled H_2_O was dropped inside away from the sample. A 300 W Xe source from LOT-Quantum Design equipped with an AM1.5G filter was adjusted so that the surface of the sample was subjected to 1 sun (100 mW cm^–2^). Once set up, the photoreactor was briefly evacuated by using a vacuum pump and then refilled with CO_2_. The system was further purged with CO_2_ at 10 mL min^–1^ for 15 min. A relative humidity of 55% was measured at the outlet of the photoreactor just before the reaction using a LANDTEK HT6292 digital dew point meter. For the reaction, the inlet and outlet of the reactor were closed and left for another 15 min to equilibrate. Finally, the CO_2_ reduction experiment was run for 1 h at 1 sun. The amount of water, reaction time, and sample preparation method were optimized for pure bulk g-C_3_N_4_, and the results are illustrated in Figure S1. To measure the emerged gases, CO_2_ was used as a carrier gas to reach a Shimadzu QP 2020NX gas chromatograph unit with a barrier ionization discharge (BID) detector and mass spectrometer (MS). The apparent quantum efficiency of the catalyst was measured in the same setup and at the same experimental conditions. Instead of illuminating using the 300 W Xe source, a 365 nm LED light was used. To ensure that the products were formed by the photocatalytic reduction of CO_2_, control experiments were conducted to ensure that no other species in the reactor affected the photocatalytic results. The control tests were conducted (1) by physically mixing g-C_3_N_4_ with Cs_3_Bi_2_Br_9_, (2) in helium (He) with H_2_O, (3) in He without H_2_O, (4) in the absence of catalyst (only quartz filter paper), and (5) in the dark. In addition, the isotope labeled ^13^CO_2_ (BOC, >98% atom ^13^CO_2_ compared to ^12^CO_2_, >99%) was used to verify that the production was due to the photocatalytic conversion of CO_2_. The reactor was purged with ^13^CO_2_ for 15 min at 10 mL min^–1^ and irradiated for 5 h at 1 bar pressure with 40 μL H_2_O drops inside the reactor. The gases were analyzed by a Shimadzu GCMS-QP2020 NX gas chromatograph equipped with an Rt-Q-Bond Plot Column and molecular sieve columns (SH-Msieve 5A plot, 30 m, 0.32 mm ID, 30 μm, Shimadzu) in series.

### Photocurrent Measurement

Thin films were prepared by spin coating fluorine-doped tin oxide (FTO) coated glass with a 50 mg mL^–1^ dispersion of the as-prepared powders in anhydrous 2-propanol. First, FTO-coated glass slides of 2.5 × 3 cm^2^ were cleaned in three consecutive stages through ultrasonication using distilled water (DI) and Hellmanex III (2% aqueous solution), 2-propanol, and DI water for 10 min each. Then, the prepared dispersion was spin-coated five times on the FTO coated glass at a speed of 1000 rpm for 20 s. The glass was placed on a hot-plate to dry at 70 °C for 30 min. Photoelectrochemical (PEC) measurements were conducted using a three-electrode cell setup. The electrolyte was a solution of 0.1 M TBAPF_6_ in anhydrous acetonitrile. The prepared films functioned as a working electrode with an active surface area of 0.28 cm^2^ with a Pt wire counter electrode and a nonaqueous Ag/Ag+ reference electrode filled with 0.01 M AgNO_3_ and 0.1 M TBAPF_6_ in acetonitrile. Simulated sunlight (100 mW cm^–2^) from a Lot Quantum Design xenon lamp source equipped with an AM 1.5G filter was used to illuminate the films, while a Compactstat potentiostat from Ivium Technologies was used to control the potential of the working electrode.

## Results and Discussion

### Materials Synthesis and Characterization

**Figure 1:**
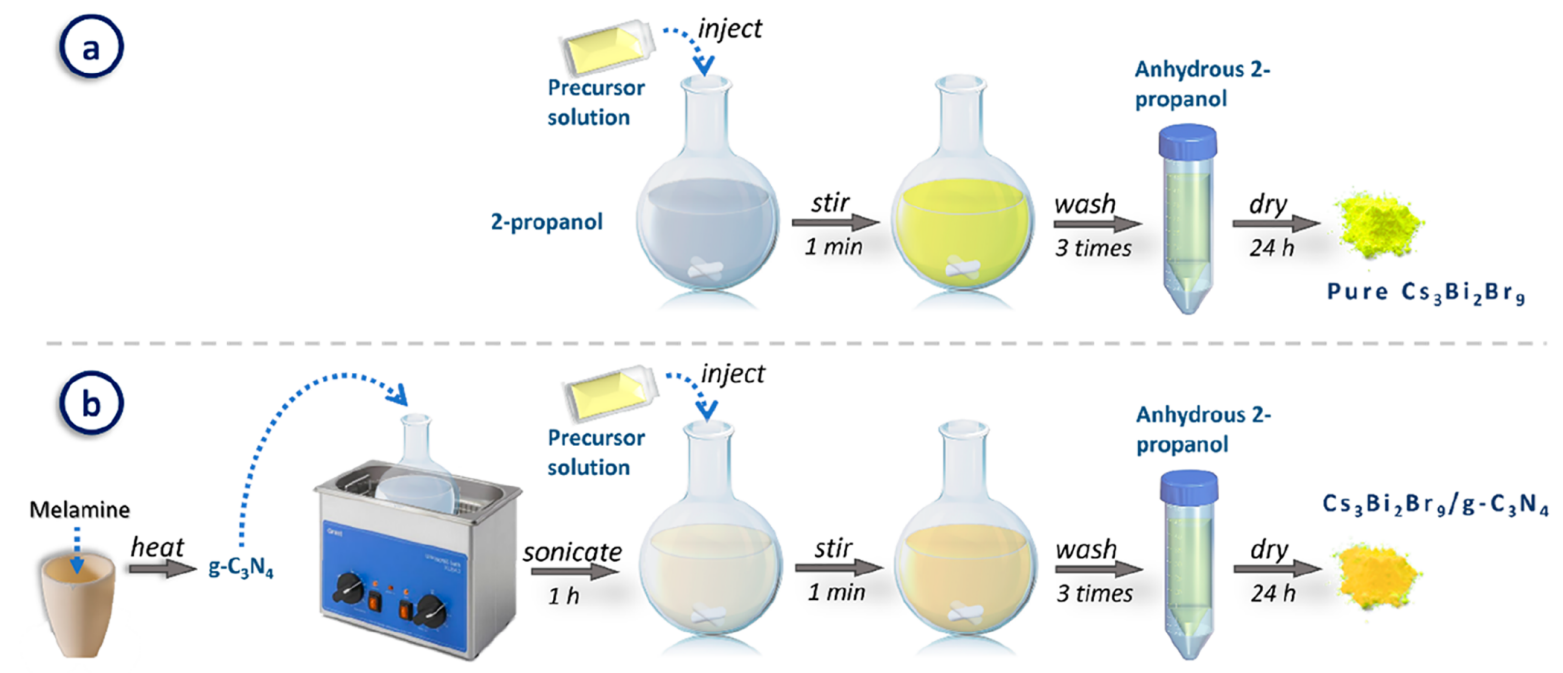
Schematic representation of the synthesis process of (a) pure Cs_3_Bi_2_Br_9_ and (b) Cs_3_Bi_2_Br_9_/g-C_3_N_4_ composites.

Pure Cs_3_Bi_2_Br_9_ was synthesized using a straightforward antisolvent crystallization process which entails the swift injection of the precursor solution (CsBr, BiBr_3_, dimethyl sulfoxide) into the antisolvent, isopropyl alcohol, for precipitation (Figure 1a). For the synthesis of the Cs_3_Bi_2_Br_9_/g-C_3_N_4_ composites, a preceding step was added where the required amount of g-C_3_N_4_ was dispersed in the antisolvent before the Cs_3_Bi_2_Br_9_ crystallization process (Figure 1b).

**Figure 2:**
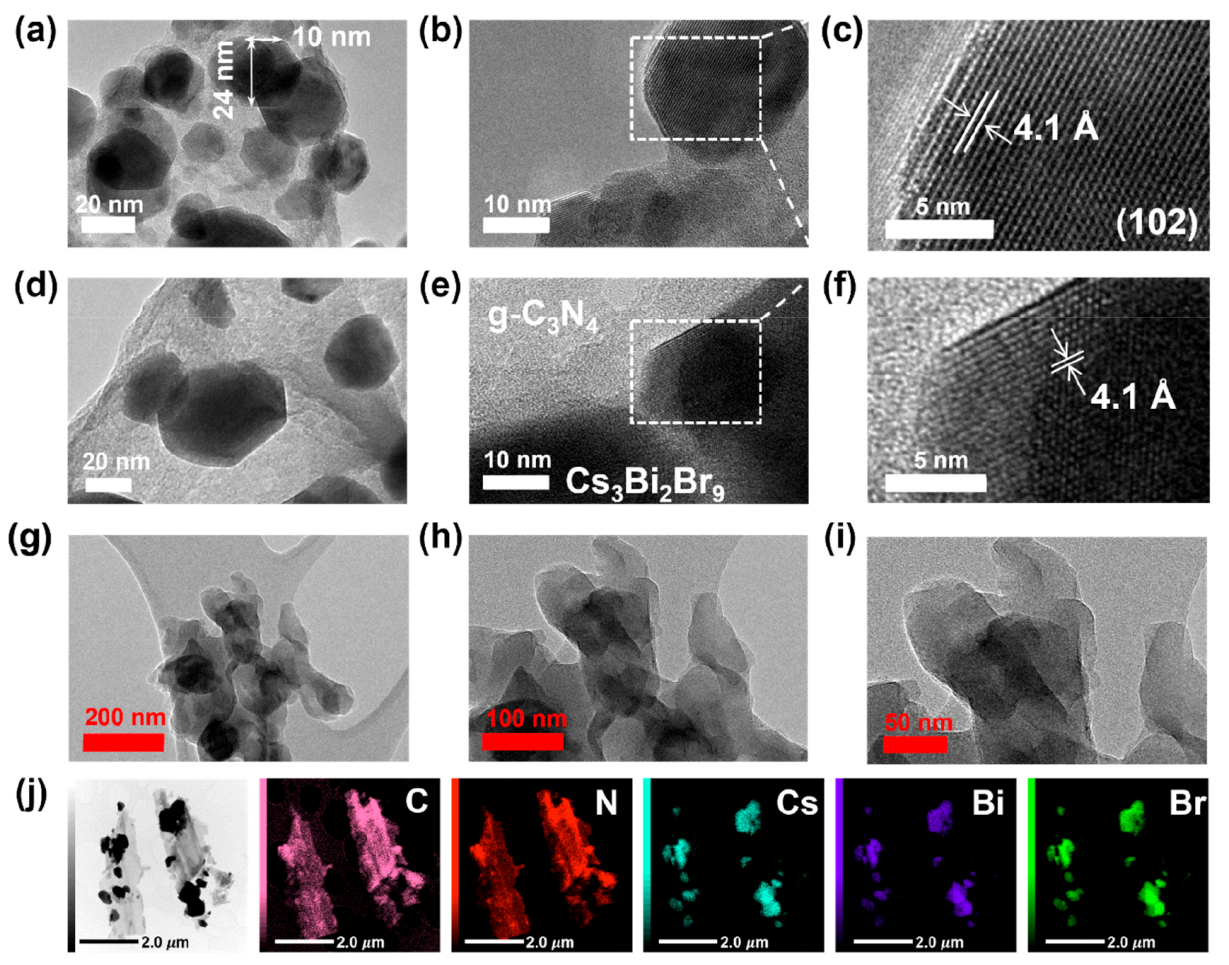
TEM micrographs of (a, b) pure Cs_3_Bi_2_Br_9_, (d, e) 40 wt % Cs_3_Bi_2_Br_9_/g-C_3_N_4_ composite, and (g, i) pure g-C_3_N_4_. HRTEM micrographs of (c) pure Cs_3_Bi_2_Br_9_ and (f) 40 wt % Cs_3_Bi_2_Br_9_/g-C_3_N_4_. (j) TEM micrograph coupled with EDX mapping for the 40 wt % Cs_3_Bi_2_Br_9_/g-C_3_N_4_ sample showing scans for C, N, Cs, Bi, and Br.

To visualize and understand the morphology and crystal structure of the pure semiconductors and Cs_3_Bi_2_Br_9_/g-C_3_N_4_ composites, TEM and high-resolution TEM were used. The as-synthesized Cs_3_Bi_2_Br_9_ formed hexagonal nanocrystals with an easily identifiable lattice distance of 4.1 Å, assigned to the (102) plane (Figure 2a–c). On the other hand, pure g-C_3_N_4_ showed an irregular morphology with an amorphous structure (Figure 2h–i).^20^ TEM micrographs of the 40 wt % Cs_3_Bi_2_Br_9_/g-C_3_N_4_ composite confirmed a homogeneous distribution of the hexagonal crystals on the micrometric-sized amorphous g-C_3_N_4_ (Figure 2g). The synthesis process did not affect the crystal structure of Cs_3_Bi_2_Br_9_ since the lattice distance of the crystals remained 4.1 Å (Figure 2d–f). Additional TEM micrographs coupled with EDX mapping of the 40 wt % Cs_3_Bi_2_Br_9_/g-C_3_N_4_ composite were taken to identify the distribution of the main atoms constituting the two semiconductors (Figure 2j). Two relatively large particles with a length and width of approximately 3 and 1 μm, respectively, mainly composed of C and N were observed and assigned to the sheet-like g-C_3_N_4_. On the surface, irregular clusters made of Cs, Bi, and Br atoms confirmed the formation of Cs_3_Bi_2_Br_9_ crystal clusters dispersed on the surface of g-C_3_N_4_.

**Figure 3:**
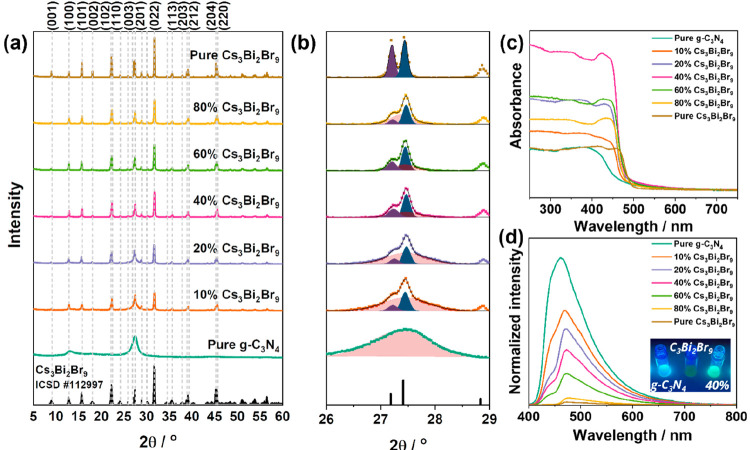
(a) XRD spectra of pure g-C_3_N_4_ and Cs_3_Bi_2_Br_9_ (ICSD #112997)^22^ in comparison to the formed composites with a (b) zoomed-in diffractogram of the region 26–29° with Gaussian fitting of the (201) plane in purple, (112) plane in navy of Cs_3_Bi_2_Br_9_, and the (002) plane of g-C_3_N_4_ in red. (c) UV–visible absorbance spectra of the different samples. (d) Steady-state PL spectra of the samples with an excitation wavelength λ_*ex*_ = 380 nm laser with an inset image of the pure semiconductors and the 40% Cs_3_Bi_2_Br_9_/g-C_3_N_4_ composite. Spectra were normalized to their weight (pure semiconductors) or the individual weight of g-C_3_N_4_ in the composites.

XRD measurements were carried out to study the crystal structures of the two materials (Figure 3a). g-C_3_N_4_ had broad peaks in agreement with its known stacked structure of sp^2^-hybridized nitrogen-substituted graphene.^21^ The broad peaks were present at 13.1 and 27.4° corresponding to the (100) and (002) planes, respectively. On the other hand, Cs_3_Bi_2_Br_9_ had narrower diffraction peaks in agreement with a crystalline particle structure. The main diffraction peaks were centered at 13.1, 16.1, 22.4, 27.2, 27.4, 31.9, 35.8, 39.4, and 45.5° (2θ) corresponding to the planes (100), (101), (102), (201), (112), (202), (212), and (220), respectively. The diffractogram was matched with patterns reported in literature (ICSD #112997),^22^ and it belonged to the trigonal *P*3̅*m*1 space group.^23^ XRD diffractograms of the composites showed convoluted peaks out of broad diffraction peaks of g-C_3_N_4_ and narrower peaks of Cs_3_Bi_2_Br_9_ in their respective proportion, confirming the formation of composites (Figure 3b).^17^ Applying a Gaussian deconvolution of the peaks between 26 and 29°, we were able to study the (002) plane of g-C_3_N_4_ as well as the (201) and (112) planes of Cs_3_Bi_2_Br_9_. The characteristic (002) peak of pure g-C_3_N_4_ decreased in intensity with the increase in Cs_3_Bi_2_Br_9_ and kept its position centered at 27.4°, suggesting no changes to the g-C_3_N_4_ crystallinity. On the other hand, the area of the (112) peak of Cs_3_Bi_2_Br_9_ increased with content from 387.6 (10 wt % Cs_3_Bi_2_Br_9_) to 590.6 au (pure Cs_3_Bi_2_Br_9_). The deconvolution also highlighted the preferential growth of the (112) plane rather than the (201) plane of Cs_3_Bi_2_Br_9_ during the crystallization process in the presence of g-C_3_N_4_. Full details of the parameters obtained from the peak deconvolutions are shown in Table S1. The diffraction peak at 22.4° assigned to the (102) plane of Cs_3_Bi_2_Br_9_ corresponds to the lattice fringes of 4.1 Å observed in the TEM micrograph in Figure 2c.

Ultraviolet–visible (UV–vis) spectroscopy conducted in diffuse reflectance mode was performed on the two semiconductors (Figure 3c). It was observed that bulk g-C_3_N_4_ and Cs_3_Bi_2_Br_9_ absorbed similar wavelengths of light with absorption edges at around 463 and 493 nm, respectively. Measuring the absorbance of the composites in comparison to the pure semiconductors showed a higher absorbance between 300 and 450 nm with a slight redshift for the composites.^24^ In particular, the 40 wt % Cs_3_Bi_2_Br_9_/g-C_3_N_4_ composite demonstrated the highest light absorbance, suggesting that this composition forms a heterostructure where the two semiconductors complement each other in terms of light harvesting ability, which is important for the photogeneration of charges. Steady-state PL spectra of the pure samples were measured upon 380 nm excitation and normalized to their weight (Figure 3d). Pure g-C_3_N_4_ showed PL emission with a maximum at 461.4 and a shoulder around 440 nm. Cs_3_Bi_2_Br_9_ instead emits PL centered at 471.4 nm and of lower intensity, probably due to nonradiative recombination. The PL emission of both g-C_3_N_4_ and Cs_3_Bi_2_Br_9_ is assigned to their bandgaps. The PL spectra of the composites, normalized to the weight of g-C_3_N_4_ in the composite, the most PL emitter, showed the same wide PL emission with a maximum around 460–470 nm but the intensity decreased with the addition of Cs_3_Bi_2_Br_9_. The decrease in intensity was more pronounced at wavelengths around 440 nm (2.8 eV) which corresponds to the direct bandgap of g-C_3_N_4_ (Figure S2).^25^ The observations in the PL spectra agree with an optical picture of the samples under UV 365 nm LED excitation that shows a clear color change from blue to aqua on pure g-C_3_N_4_ and 40% Cs_3_Bi_2_Br_9_/g-C_3_N_4_ and negligent luminescence in pure Cs_3_Bi_2_Br_9_. The PL decrease in intensity in the composites with Cs_3_Bi_2_Br_9_ is assigned to the charge transfer of photoinduced electrons and holes between g-C_3_N_4_ and Cs_3_Bi_2_Br_9_. In addition, time-resolved PL was performed on the samples, and the findings are displayed in Figure S3 and summarized in Table S2. It was observed that the incorporation of Cs_3_Bi_2_Br_9_ into the composite decreased the PL lifetimes in comparison to the pure g-C_3_N_4_.

To further analyze the surface functional groups on the pure semiconductors as well as the composites, FTIR and Raman spectroscopy were performed. FT-IR was used mainly to assess organic g-C_3_N_4_ species as shown in Figure S4a. Pure g-C_3_N_4_ had a large, yet narrow peak centered at 3126 cm^–1^ assigned to N–H stretching vibrations. The sharp peaks in the regions of 1635–1444 cm^–1^ and 1392–1240 cm^–1^ are assigned to C=N and aromatic C–N stretching vibrations, respectively. Next, the sharp peak at 805 cm^–1^ was assigned to the tri-s-triazine ring vibrations. The absence of a sharp peak at around 1730 cm^–1^ proves the absence of C=O stretching and confirms that there was no surface oxidation of g-C_3_N_4_ as also concluded from the XPS analysis. These observations agree with existing literature.^21,26^ It was evident that the decrease in the g-C_3_N_4_ content led to a decrease in the intensity of all peaks on the spectrum. Raman spectra collected using a laser light at a 785 nm wavelength demonstrated the presence of 5 main peaks for Cs_3_Bi_2_Br_9_ (Figure S4b). Two peaks at higher shifts of 166 and 190 cm^–1^ were attributed to the A_1g_ and E_g_ modes of the Bi–Br vibrations. The absence of any peaks between 130 and 160 cm^–1^ confirms that no secondary double Cs_3_BiBr_6_ phase was formed during the synthesis process.^27^ The findings are in good agreement with similar reported Raman spectra of Cs_3_Bi_2_Br_9_.^28,29^ The Raman spectra showed an increase in peak intensity with an increase in Cs_3_Bi_2_Br_9_ ratio for the composite, in agreement with the Bi–Br content. The composition of each of the samples was confirmed using CHN elemental analysis, as reported in Table S3.

**Figure 4:**
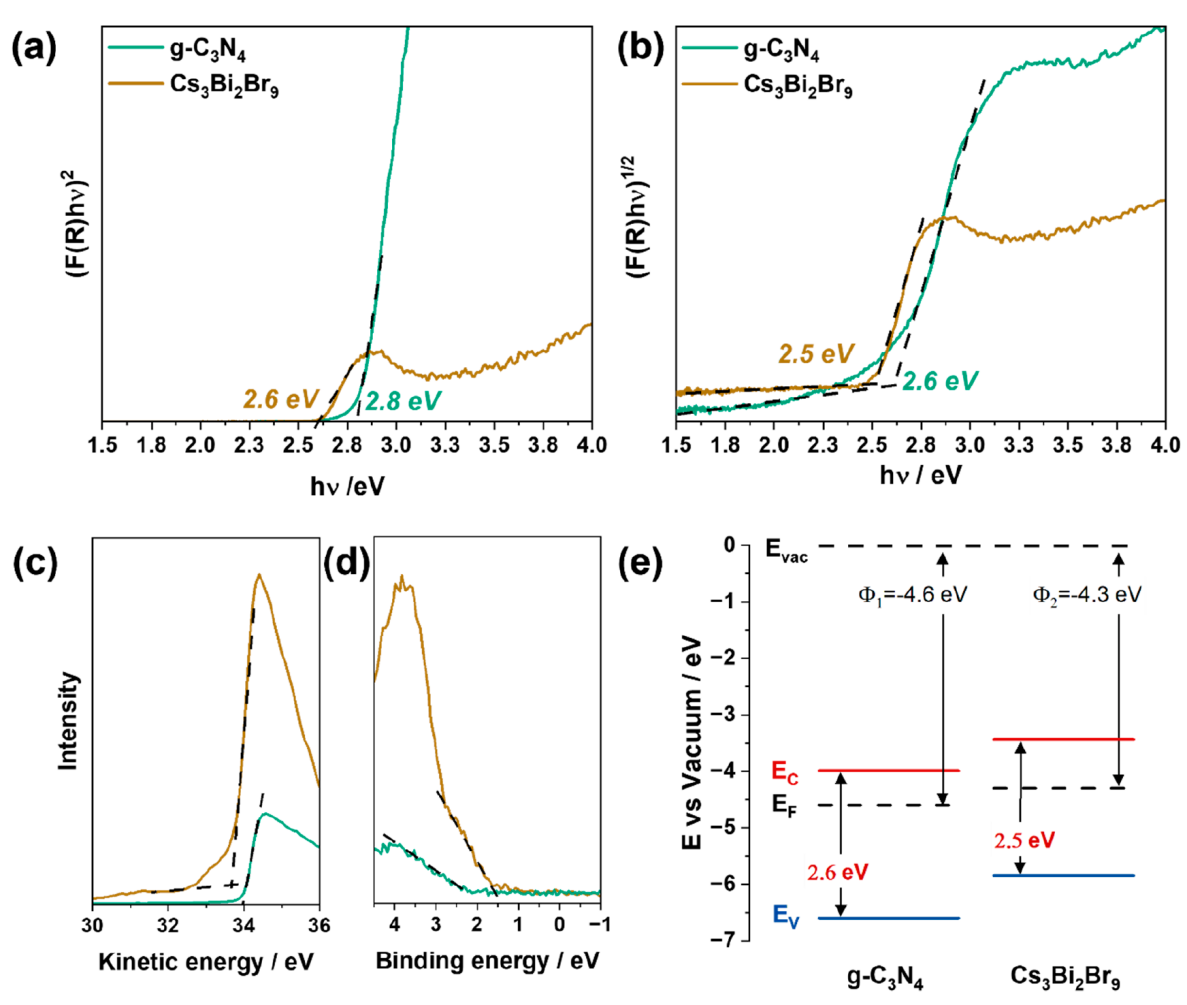
Tauc plots of the two semiconductors for bandgap analysis assuming (a) direct and (b) indirect behavior. (c) Work function and (d) valence band edge measurements using valence band XPS for g-C_3_N_4_ and Cs_3_Bi_2_Br_9_. (e) Conduction and valence band edge positions and Fermi levels of the two pure semiconductors.

Using the Kubelka–Munk function, UV–vis reflectance spectra were converted to Tauc plots, where the initial linear region of the curves was extrapolated to identify the band gap of the two semiconductors. As a result, a direct band gap of 2.8 eV (g-C_3_N_4_) and 2.6 eV (Cs_3_Bi_2_Br_9_) as well as an indirect bandgap of 2.6 eV (g-C_3_N_4_) and 2.5 eV (Cs_3_Bi_2_Br_9_) were measured (Figure 4a,b) and compared to the literature.^25,30,31^ In addition, XPS measurements conducted with a negative applied bias (−29.4 eV) were performed to measure the work function (Figure 4c). As the work function represents the minimum energy required to remove an electron from the sample to the vacuum level, it was used to situate the Fermi levels of the samples at −4.6 eV (g-C_3_N_4_) and −4.3 eV (Cs_3_Bi_2_Br_9_) (Figure 4c). Valence-band XPS measurements were taken to estimate the energy between the Fermi level and the valence band edge of each semiconductor (Figure 4d). Finally, when the findings were coupled with the calculated band gap, the energy diagrams for g-C_3_N_4_ and Cs_3_Bi_2_Br_9_ were constructed (Figure 4e). Based on the band edge alignment, the two semiconductors can either form a type-II or a direct Z-scheme heterojunction. In a type-II heterojunction, electrons would travel from the higher conduction band of Cs_3_Bi_2_Br_9_ (−3.44 eV vs vacuum) to the conduction band of g-C_3_N_4_ (−4.28 eV vs vacuum), while holes travel the opposite way in their valence bands. In a direct Z-scheme heterojunction, electrons would accumulate in the Cs_3_Bi_2_Br_9_ conduction band and holes in the valence band of g-C_3_N_4_ while recombination occurs between electrons in the g-C_3_N_4_ conduction band and holes in the Cs_3_Bi_2_Br_9_ valence band. These configurations are more favorable than a type-I heterojunction, where electrons and holes from both semiconductors would accumulate and recombine further in the semiconductor with a smaller band gap. Importantly, since the Fermi level of Cs_3_Bi_2_Br_9_ was shallower, the formation of a direct Z-scheme configuration would be favored over a type-II heterojunction. In such configuration, g-C_3_N_4_ would experience downward band bending and Cs_3_Bi_2_Br_9_ upward band bending, and the charge separation will direct electrons and holes toward the furthest apart conduction and valence band edges increasing their driving force for reduction and oxidation reactions, respectively.

**Figure 5:**
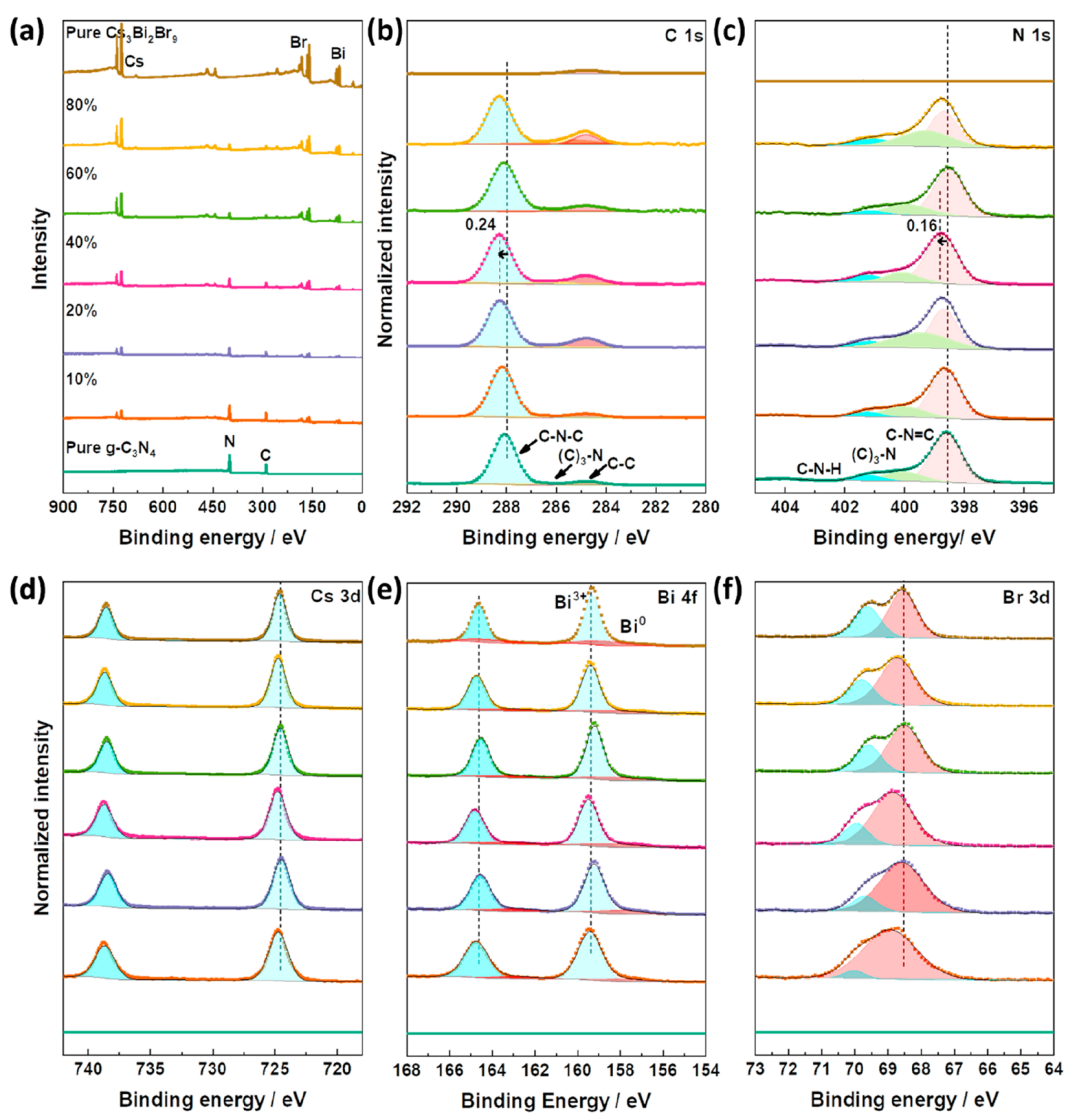
XPS (a) survey scans with normalized scans of (b) C 1s, (c) N 1s, (d) Cs 3d, (e) Bi 4f, and (f) Br 3d of the pure semiconductors and their composites

XPS was conducted on the pure materials and composites to further understand the surface composition (Figure 5). A survey scan of bulk g-C_3_N_4_ showed C and N as the main constituting atoms. In more detail, the deconvolution of peaks in a C 1*s* scan showed the presence of two main carbon species. The peak with the lowest binding energy (BE) was corrected to 284.8 eV representing adventitious carbon forming C–C or graphitic C=C bonds. The higher-intensity peak centered at 288.2 eV is assigned to N–C=N bonds within the aromatic rings of g-C_3_N_4_ (sp^2^ hybridization). In addition, a N 1*s* scan showed three nitrogen species at BE values of 398.7, 340, and 401.3 eV attributed to pyridinic C–N=C, graphitic N–(C)_3_, and pyrrolic C–N–H bonding, respectively. For Cs_3_Bi_2_Br_9_, the survey scan showed the presence of Cs, Bi, and Br species. A doublet with peaks at 724.6 and 738.5 eV was observed in the Cs 3*d* scan ascribed to Cs 3*d*_5/2_ and Cs 3*d*_3/2_, respectively. Similarly, a Bi 4f scan demonstrated the presence of a Bi^3+^ species with a doublet at 159.4 and 164.7 eV with a spin–orbit separation of 5 eV indicating the 3^+^ oxidation state and a small doublet Bi (0) at 159.31 and 164.63 eV. Finally, a Br 3*d* scan was deconvoluted into only two peaks at 68.7 and 70 eV attributed to the presence of Br in the Br 3*d*_5/2_ and Br 3*d*_3/2_, respectively. In general, comparing the peak intensities of the different samples shown in Figure S5, a clear increase in the Cs_3_Bi_2_Br_9_ peaks (Cs 3*d*, Bi 4*f*, and Br 3*d*) was observed with increasing crystal content with respect to g-C_3_N_4_. The spectra were normalized to clearly distinguish peak shifts in Figure 5 with a detailed summary of the deconvoluted peak locations in Table S4. Importantly, there was a chemical shift for C 1*s* and N 1*s* to higher binding energies, which is here assigned to downward band bending in the g-C_3_N_4_ when Cs_3_Bi_2_Br_9_ is grown on it.^32^ This agrees well with the energy diagram in Figure 4e, which points at the formation of a direct Z-scheme configuration upon contact between semiconductors in which g-C_3_N_4_ will experience interfacial downward band bending.

### Photocatalytic Performance

**Figure 6:**
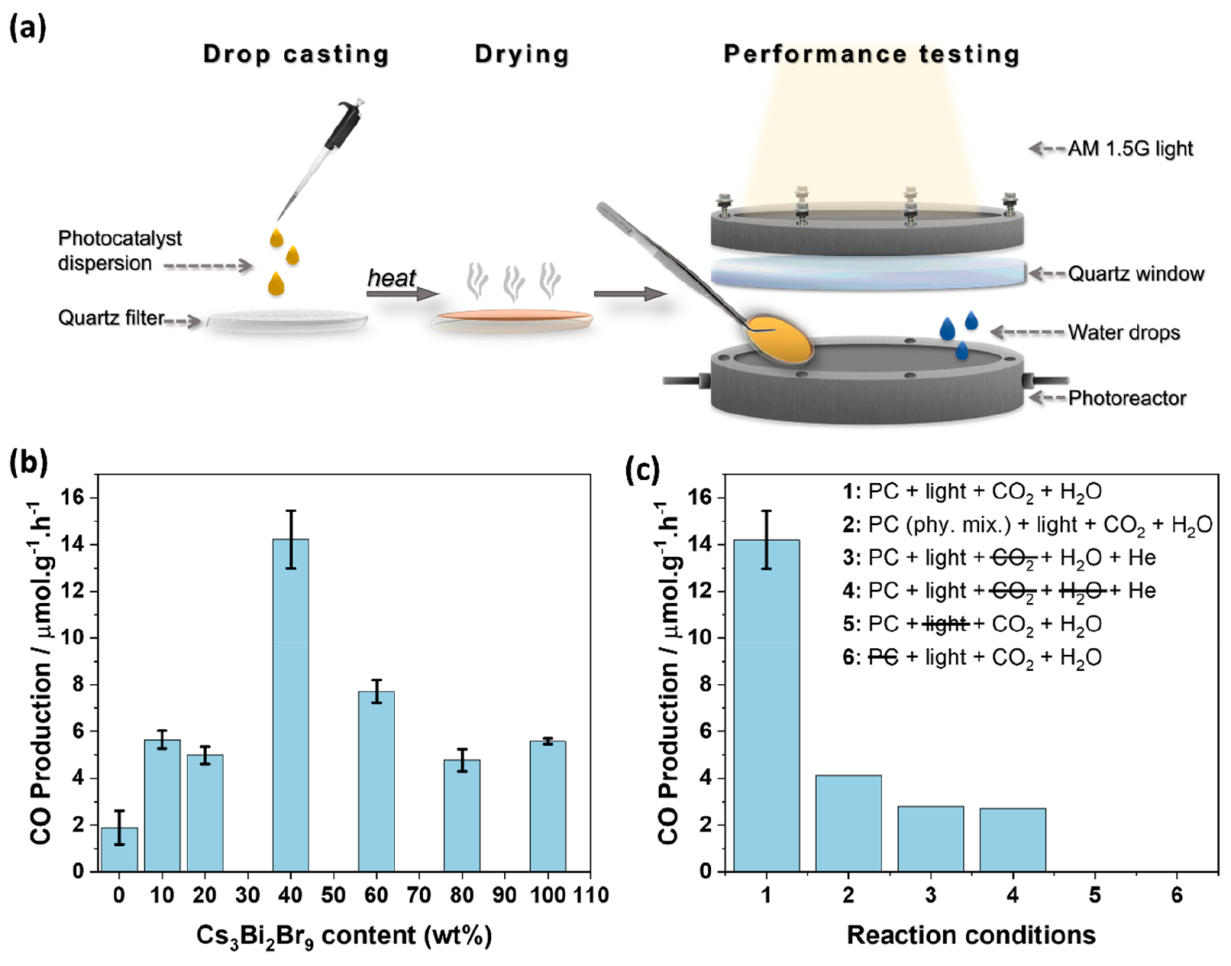
(a) Schematic representation of the sample preparation for photocatalytic performance testing. (b) Photocatalytic CO_2_ reduction rate to CO at different Cs_3_Bi_2_Br_9_ loadings on g-C_3_N_4_. (c) Summary of production rates of 40% Cs_3_Bi_2_Br_9_/g-C_3_N_4_ at different control reaction conditions.

The photocatalytic reduction of CO_2_ using 40 μL of water as a proton donor was performed for 1 h under 1 sun and the results are illustrated in Figure 6a. It was observed that the pure semiconductors as well as the synthesized composites were selective to CO production under the chosen reaction conditions. As reported in literature, g-C_3_N_4_ and Cs_3_Bi_2_Br_9_ photocatalysts used for gas-phase photocatalytic CO_2_ reduction often demonstrate the kinetically favorable production of CO over CH_4_ or H_2_.^12,33−36^ Pure g-C_3_N_4_ showed the production of CO at a rate of 1.89 (±0.72) μmol CO g^–1^ h^–1^ while pure Cs_3_Bi_2_Br_9_ showed a CO production of 5.58 (±0.14) μmol CO g^–1^ h^–1^. As soon as 10 wt % Cs_3_Bi_2_Br_9_ was added to g-C_3_N_4_, the production increased by approximately 44% to 5.65 (±0.38) μmol of CO g^–1^ h^–1^ compared with g-C_3_N_4_. As the ratio of Cs_3_Bi_2_Br_9_ to g-C_3_N_4_ increased, a maximum production of 14.22 (±1.24) μmol of CO g^–1^ h^–1^ was achieved at 40 wt % Cs_3_Bi_2_Br_9_. 40 wt % was then the optimum loading of Cs_3_Bi_2_Br_9_ needed to achieve an optimal heterojunction with g-C_3_N_4_ for photocatalytic CO_2_ reduction to CO. A further increase in Cs_3_Bi_2_Br_9_ content suppressed the photocatalytic activity gains due to probably the inhomogeneous distribution of the crystals on the g-C_3_N_4_ surface hindering light absorption by g-C_3_N_4_ and the impaired balance of holes and electrons in the Z-scheme charge transfer mechanism. Overall, the production of the 40% Cs_3_Bi_2_Br_9_/g-C_3_N_4_ composite on an electron basis was 28.43 μmol e^–^ g^–1^ h^–1^; 2.5 times higher than that of pure Cs_3_Bi_2_Br_9_ (11.12 μmol e^–^ g^–1^ h^–1^) or 7.5 times higher than that of pure g-C_3_N_4_ (3.78 μmol e^–^ g^–1^ h^–1^). To ensure that the production was indeed CO_2_ photocatalytic reduction, a series of control tests was performed on pure Cs_3_Bi_2_Br_9_, pure g-C_3_N_4_, as well as the composites in the absence of light, the catalyst, CO_2_, H_2_O, and CO_2_+H_2_O (Figure 6c). Physically mixing g-C_3_N_4_ with Cs_3_Bi_2_Br_9_ at a 40 wt % ratio produced approximately 4.13 μmol CO g^–1^ h^–1^, comparable with the values obtained when testing the pure semiconductors. This physical mixture results in 70% less production rate than the one obtained after performing the antisolvent crystallization of Cs_3_Bi_2_Br_9_ directly on the g-C_3_N_4_, further highlighting the enhanced charge separation between the two semiconductors. On the other hand, control tests performed in the absence of either one or both, CO_2_ and H_2_O, showed more than 80% decrease in production. Any residual production of CO was due to the photocatalytic degradation of adventitious carbon found on the surfaces of the samples. Finally, in the absence of light or photocatalyst, no CO evolved, confirming that the reported CO production was photocatalytic. Isotope labeled tests were conducted by purging the reactor with ^13^CO_2_ and irradiating for 5 h. The results showed the evolution of ^29^CO and confirm the photocatalytic reduction of CO_2_ (Figure S6). The apparent quantum efficiency (AQE) of the 40 wt % Cs_3_Bi_2_Br_9_ composite was calculated to be around 0.0015% by performing the reaction under a 365 nm monochromatic light at 100 mW cm^–2^ intensity. Details of the calculation steps are shown in the Supporting Information. Table S5 summarizes the obtained photocatalytic activity with respect to similar composites prepared in literature.

**Figure 7:**
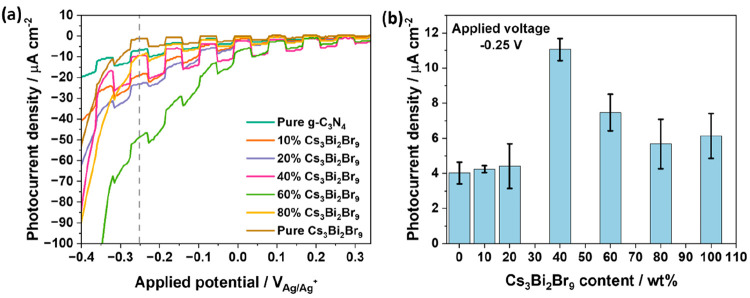
(a) Photocurrent density–voltage curves of pure g-C_3_N_4_, Cs_3_Bi_2_Br_9_, and the composites obtained under chopped simulated-sunlight illumination of 100 mW cm^–2^ in a solution of 0.1 M TBAPF_6_ in anhydrous acetonitrile. (b) Average of three measurements of photocurrent density achieved at −0.25 V.

The photocurrents evolved from films of the pure semiconductors and the synthesized composites were tested by conducting a voltage sweep from +0.3 to −0.4 V to −0.4 V vs the Ag/AgCl reference electrode while measuring the current density under chopped illumination (100 mW cm^–2^). A three-electrode electrochemical cell was used with a solution of 0.1 M TBAPF_6_ in anhydrous acetonitrile as the electrolyte due to the instability of Cs_3_Bi_2_Br_9_ in aqueous solutions. Figure 7a illustrates their current–voltage curves. Taking a potential of −0.25 V vs Ag/AgCl as a basis for comparison between the different samples, a trend similar to the one obtained in the batch photocatalytic reaction testing (Figure 6b) was observed in photocurrent values (Figure 7b). Pure g-C_3_N_4_ and pure Cs_3_Bi_2_Br_9_ demonstrated a photocurrent density of 4.03 (±0.62) μA cm^–2^ and 6.13 (±1.2) μA cm^–2^, respectively. The highest photocurrent was obtained by the 40 wt % Cs_3_Bi_2_Br_9_ composite (11.10 ± 0.6 μA cm^–2^) with approximately 2.7 times and 1.8 times an increase in photocurrent compared with g-C_3_N_4_ and Cs_3_Bi_2_Br_9_, respectively. Higher photocurrents in the composites, especially in the 40% Cs_3_Bi_2_Br_9_ composite, indicate that composites offer superior charge separation when photoinduced charges are generated. This increased charge separation favors photocatalytic processes, since it mitigates electron–hole charge recombination.^7,37^

**Figure 8:**
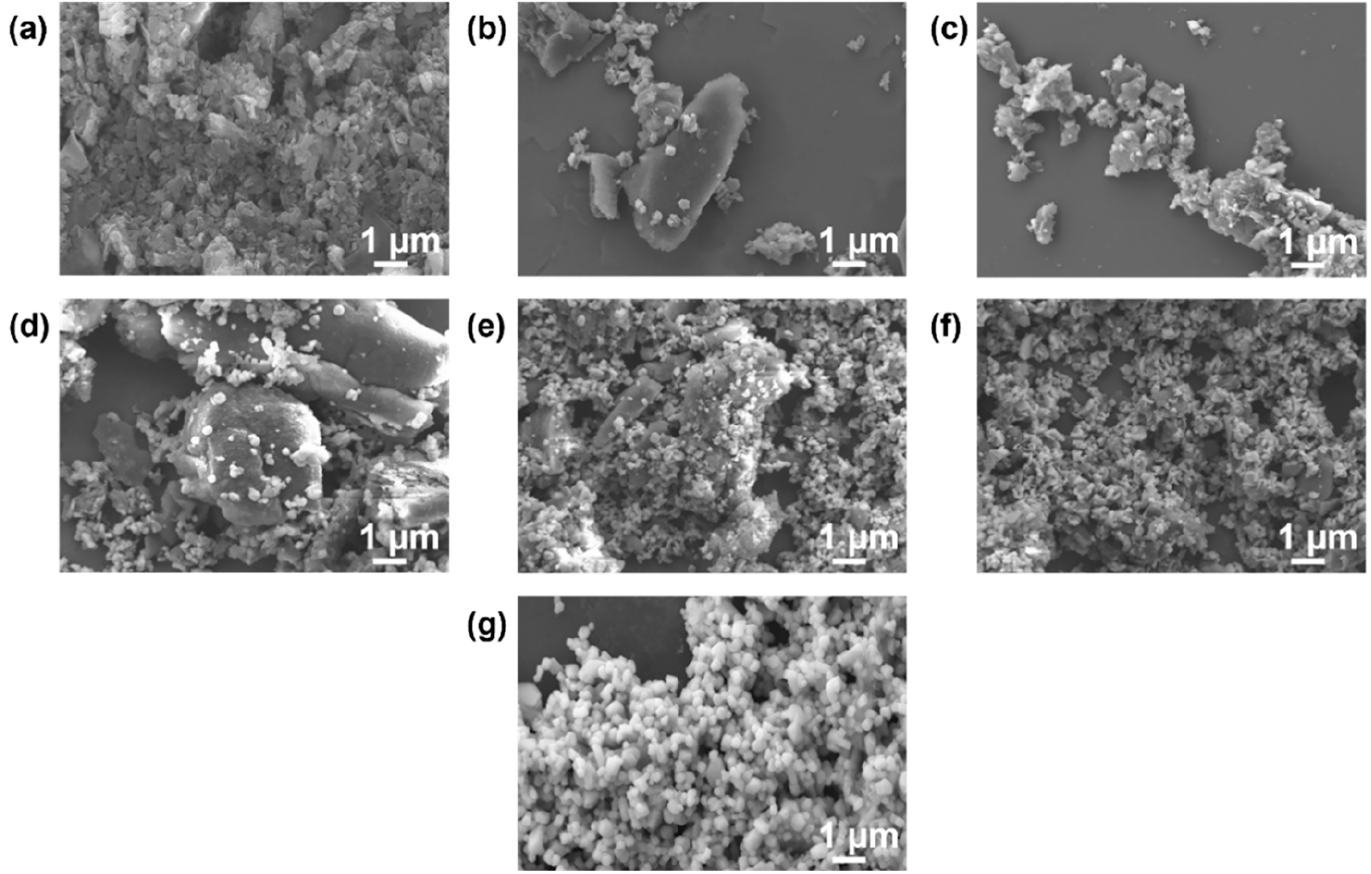
SEM micrographs of the composites with (a) pure g-C_3_N_4_ and (b) 10 wt %, (c) 20 wt %, (d) 40 wt %, (e) 60 wt %, (f) 80 wt %, and (g) 100 wt % Cs_3_Bi_2_Br_9_.

To better understand the effects of Cs_3_Bi_2_Br_9_ wt % on photocatalytic activity, SEM micrographs were taken of the different composites and are shown in Figure 8. In comparison to the morphology of pure Cs_3_Bi_2_Br_9_ shown in Figure 8g, when only 10 and 20 wt % ratios were synthesized, the morphology of the perovskite was mostly plate-like with irregular shapes and sizes (Figure 8b,c). At 40 wt % (Figure 8d), the crystal morphology resembled that of pure Cs_3_Bi_2_Br_9_. The crystals appeared dispersed on the g-C_3_N_4_ surface without forming large clusters. For higher loadings of Cs_3_Bi_2_Br_9_, larger clusters of Cs_3_Bi_2_Br_9_ were observed and less intimate contact was seen between g-C_3_N_4_ and Cs_3_Bi_2_Br_9_ (Figure 8e,f). This observation explains the decrease in photocatalytic activity through diminishing contact and charge transfer between phases.

**Figure 9:**
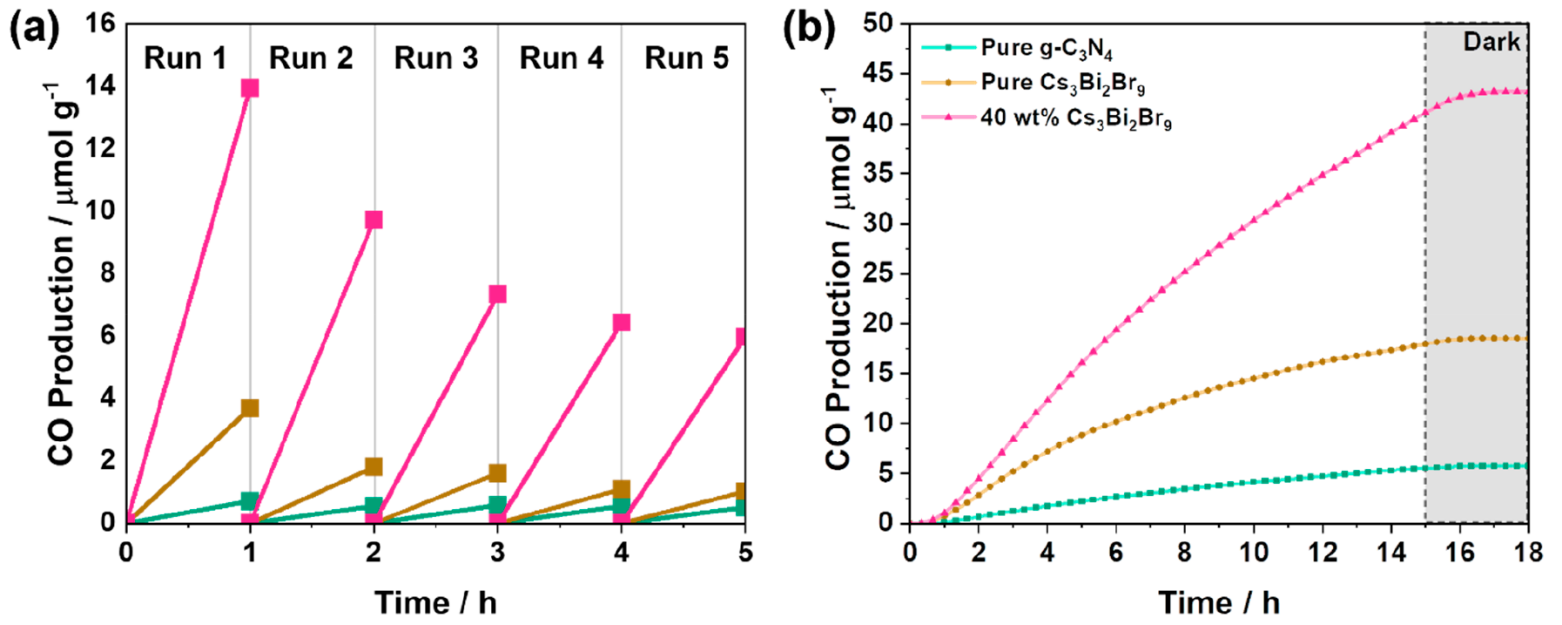
(a) Recyclability test of pure Cs_3_Bi_2_Br_9_ (brown), pure g-C_3_N_4_ (green), and 40% Cs_3_Bi_2_Br_9_/g-C_3_N_4_ (pink) over 5 consecutive cycles. (b) Cumulative CO production of continuous flow reactions performed over 15 h of illumination at a 0.5 mL/min flow of CO_2_.

To test the stability of the pure semiconductors as well as the 40 wt % Cs_3_Bi_2_Br_9_/g-C_3_N_4_ composite, the reaction was repeated on the same sample for five consecutive 1 h cycles adding fresh water drops into the reactor after each run (Figure 9a). The overall decrease in production for g-C_3_N_4_ was calculated to be approximately 28% after five cycles, with most of the loss taking place after the first 1 h. Similarly, pure Cs_3_Bi_2_Br_9_ demonstrated more than a 50% loss in production after 1 h. On the other hand, the 40 wt % Cs_3_Bi_2_Br_9_ composite showed improvement in stability in comparison to pure Cs_3_Bi_2_Br_9_ after losing approximately 30% production after the second cycle and a total of 55% after five complete cycles. A discoloration of the sample was noticed, and it was assumed that the perovskite began to degrade within 5 h of illumination. To verify this observation, continuous flow reactions were conducted for a total of 15 h of illumination at 0.5 mL min^–1^ of CO_2_ flow with measurements taken at 20 min intervals using the in-line GC system. The cumulative production of CO was calculated and is illustrated in Figure 9b for pure g-C_3_N_4_, pure Cs_3_Bi_2_Br_9_, and the 40 wt % Cs_3_Bi_2_Br_9_ composites. After 15 h of continuous illumination, the total production of CO amounted to around 10.1 μmol of CO g^–1^ for pure g-C_3_N_4_. The semiconductor required approximately 120 min to reach peak productivity, after which the concentration of CO produced per min began to decline steadily as demonstrated in Figure S7. On the other hand, pure Cs_3_Bi_2_Br_9_ was activated and reached a higher production within the first 140 min of illumination of approximately 18.6 μmol CO g^–1^ after 15 h. The 40 wt % Cs_3_Bi_2_Br_9_/g-C_3_N_4_ composite followed a similar trend and reached the highest production at 43.2 μmol CO g^–1^. The observed trend was in accordance with the batch reactions conducted for 1 h with the 40 wt % Cs_3_Bi_2_Br_9_ composite demonstrating the best performance with a 2.3 times improvement compared to pure Cs_3_Bi_2_Br_9_.

### Mechanism

**Figure 10:**
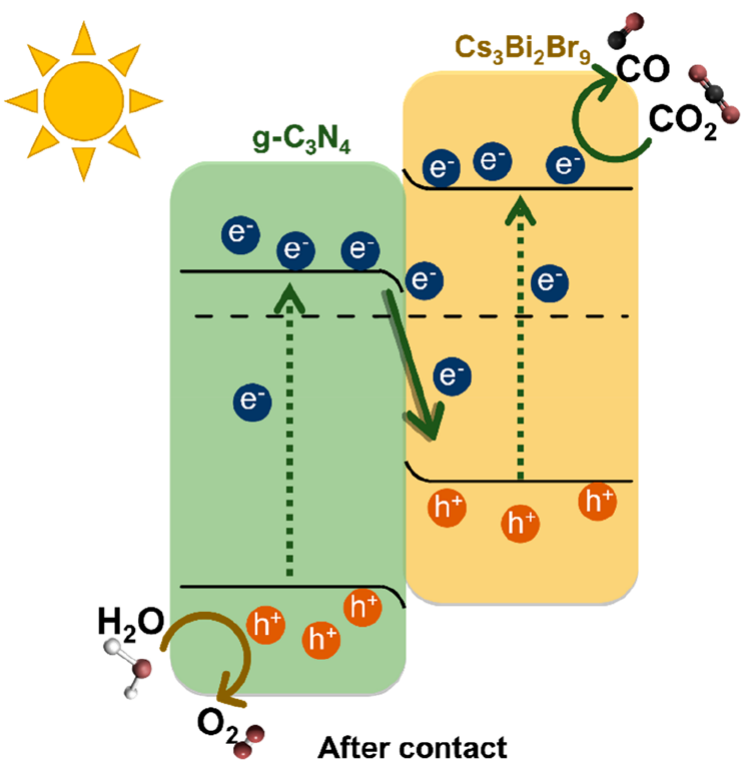
Schematic energy band diagram of the two pure semiconductors in contact forming a direct Z-scheme heterojunction.

Results of valence band XPS and UV–vis demonstrated previously in Figure 4 located the CB, VB, and Fermi levels of Cs_3_Bi_2_Br_9_ and g-C_3_N_4_ as synthesized. Based on the staggered CB and VB band-edge alignment configuration, two modes of charge transfer are possible when forming a heterojunction between the two semiconductors (Figure 10): a type-II heterojunction with electrons flowing to the g-C_3_N_4_ (reduction site) and a direct Z-scheme heterojunction with electrons accumulation on the Cs_3_Bi_2_Br_9_ (reduction site).^14,38,39^ However, when in contact, the Fermi level of the two semiconductors has to equilibrate. Based on the values gathered from valence band XPS (Figure 4) and the shallower Fermi level of Cs_3_Bi_2_Br_9_, there will be an upward band bending (i.e., electric field) formed at its interface and a downward band bending in g-C_3_N_4_. For the alignment to take place, the CB and VB of the two semiconductors will bend on the interface accordingly to form a direct Z-scheme rather than a type-II configuration, as illustrated in Figure 10.

**Figure 11:**
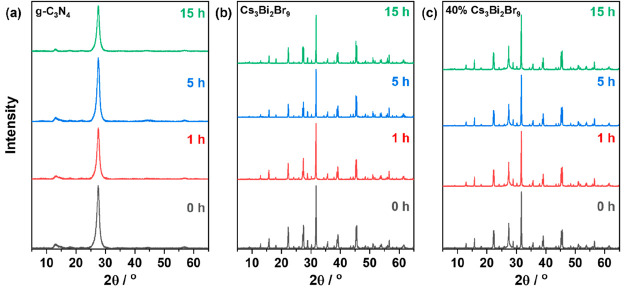
XRD diffractograms of (a) g-C_3_N_4_, (b) Cs_3_Bi_2_Br_9_, and (c) 40 wt % Cs_3_Bi_2_Br_9_ before the reaction and after 1, 5, and 15 h reactions.

To understand the mechanism of CO production, it is necessary to begin with studying the degradation process of pure materials as well as the heterojunction. As discussed in Figure 9, a decline in the photocatalytic activity of the pure semiconductors as well as the synthesized composite was observed after cycling and continuous flow testing. The decline in the activity of the different semiconductors was linked to the stability of both the organic g-C_3_N_4_ component as well as the Cs_3_Bi_2_Br_9_ crystals. One method to identify the instability in the crystal structure is by measuring the XRD diffractograms of the samples after the reaction. In the case of pure g-C_3_N_4_, postreaction characterization showed a decrease in the intensity of the (002) peak at 27.7° (2Q) in the diffractogram suggesting possible degradation of the material (Figure 11a). Similarly, XRD performed on the pure Cs_3_Bi_2_Br_9_ samples at different intervals of the 15 h reactions displayed a slight decrease in peak height and width with time compared to those of the pristine sample (Figure 11b). On the other hand, when they were put together to synthesize the 40 wt % Cs_3_Bi_2_Br_9_/g-C_3_N_4_ composite, it was observed that the decrease in peak height and width was less pronounced than in the pure g-C_3_N_4_. XPS was conducted on the samples before and after the 1 h reaction to study further any changes in the surface chemistry. XPS C 1s and N 1s peaks of pure g-C_3_N_4_ were observed to decrease slightly upon reaction (Figure S8) whereas Cs 3d, Bi 4f, and Br 3d peaks of pure Cs_3_Bi_2_Br_9_ remained almost the same (Figure S9). In the scans of the 40 wt % Cs_3_Bi_2_Br_9_/g-C_3_N_4_ composite, the signal intensity of all peaks was slightly lower upon reaction due to the higher activity these composites offer and withstand, in agreement with the decay seen in Figure 9a, but the spectra still showed the same features indicating the preservation of the composite (Figure S10).

Postreaction characterization of the samples not only gave insight into the stability of the samples but also was crucial to form an understanding of the mechanism of charge transfer between the two semiconductors. Observing the sample color changes after 1 h of illumination showed a noticeable darkening of the 40 wt % Cs_3_Bi_2_Br_9_/g-C_3_N_4_ sample in comparison to the pure semiconductors (Figure S11). After reinvestigating the Bi 4f scans of the pure Cs_3_Bi_2_Br_9_ and the 40 wt % composite, it was observed that the reduction of the Bi^3+^ of Cs_3_Bi_2_Br_9_ to Bi^0^ was observed after contacting the two materials (Figure S12). Primarily, this observation explains the decline in photocatalytic activity of the 40 wt % Cs_3_Bi_2_Br_9_/g-C_3_N_4_ since it indicates that charge accumulation has led to a reduction of the catalyst itself. The heterojunction indeed promoted charge separation between the two semiconductors. Based on the possible mechanisms of charge separation, Bi^2+^ reduction on Cs_3_Bi_2_Br_9_ can occur only if electrons could accumulate on the surface of the semiconductor. This, in turn, signifies again that a direct Z-scheme configuration was more likely to be the reason for the improvement in the efficiency of the system.

**Figure 12:**
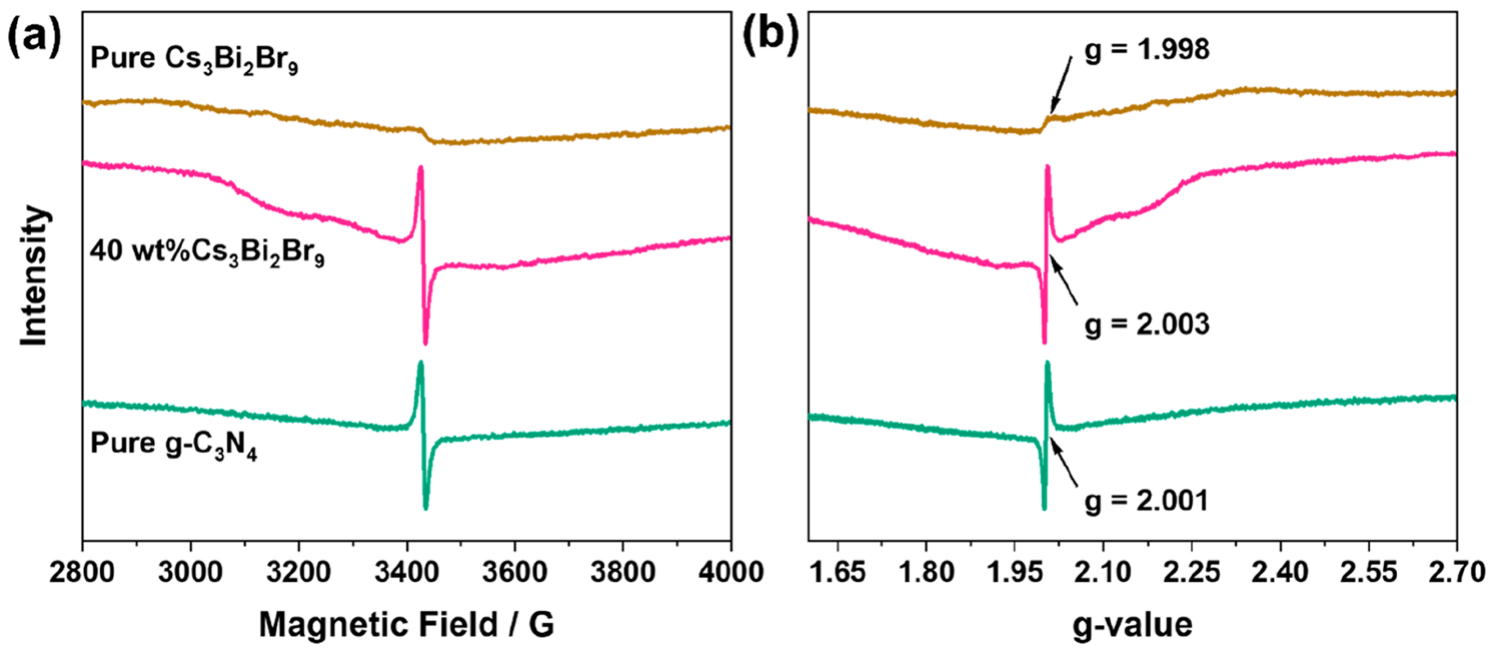
(a) EPR spectra of pure g-C_3_N_4_, pure Cs_3_Bi_2_Br_9_, and the 40 wt % Cs_3_Bi_2_Br_9_/g-C_3_N_4_ composite with (b) the calculated g-value for the samples within the studied range.

EPR measurements conducted in solid-state for the pure semiconductors and the 40 wt % composite are displayed in Figure 12. The results were used to identify the delocalization properties of the different samples. While all the samples show a similar Lorentzian line, there was a clear difference in EPR intensity between them, hinting at differences in spin–orbit coupling. For pure Cs_3_Bi_2_Br_9_, Bi–Br interactions within the crystals as well as Bi vacancies may be the main contribution to the observed paramagnetic centers demonstrating a low intensity with a signal centered at *g* = 1.998 similar to results shown in the literature.^40,41^ Pure g-C_3_N_4_ demonstrated a single, nearly isotropic, Lorentzian line at a *g*-value of 2.001 which was attributed to the unpaired electron of the carbon atoms with π-bonding within the heptazine rings.^42,43^ Similarly, the 40 wt % composite showed a signal centered at *g* = 2.003 referring to a similar spin–orbit coupling mechanism. However, the intensity of the signal was higher than that of the two pure semiconductors, which hints at an improved electron transport within the composite.

**Figure 13:**
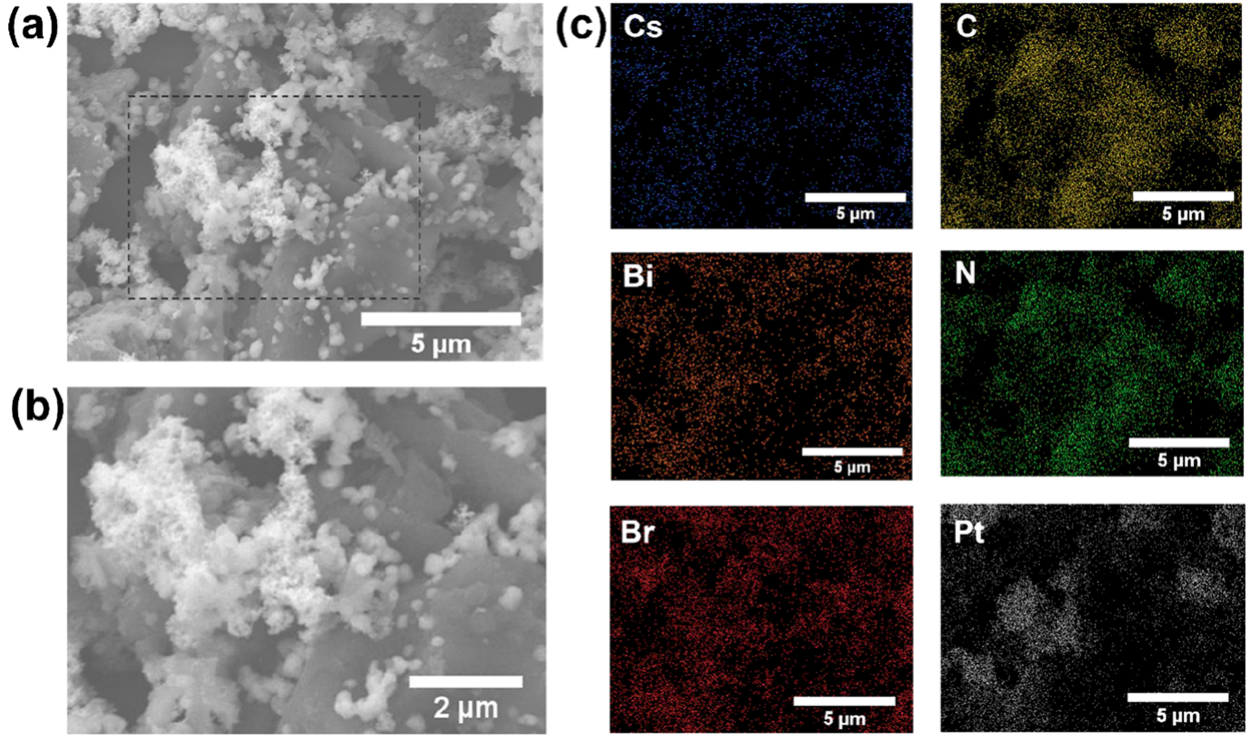
(a) SEM micrograph of 40 wt % Cs_3_Bi_2_Br_9_/g-C_3_N_4_ after the Pt photodeposition process with a (b) zoomed in section on the area with observed Pt clusters. (c) EDX mapping of Cs, Bi, Br, C, N, and Pt elements.

To further validate the formation of a direct Z-scheme heterojunction, we attempted to photodeposit platinum (Pt) from H_2_PtCl_6_·(H_2_O)_6_ dissolved in anhydrous isopropanol on the 40 wt % Cs_3_Bi_2_Br_9_/g-C_3_N_4_ composite. Although Pt can be deposited onto both the Cs_3_Bi_2_Br_9_ and g-C_3_N_4_, it is highly likely that it deposits in more abundance on the semiconductor with a higher electron density upon charge separation, which would be Cs_3_Bi_2_Br_9_ in a Z-scheme configuration (Figure 10).^23^ To test the theory, the composite was dispersed in a solution of the platinum precursor in anhydrous isopropyl alcohol. The solution was illuminated with an AM 1.5 G-filtered xenon lamp for 1 h, after which the sample was washed and collected for further analysis. SEM images of the samples (Figure 13a,b) showed a similar morphology of g-C_3_N_4_ sheets with Cs_3_Bi_2_Br_9_ crystals as the ones previously displayed in Figure 8. Figure 13b clearly displays the presence of metallic clusters in the nanometric range mainly centered on top of the Cs_3_Bi_2_Br_9_ crystals. A detailed EDX atomic scan allows identification of the areas with high density of carbon and nitrogen atoms indicating g-C_3_N_4_ as well as the inorganic species for Cs_3_Bi_2_Br_9_. Platinum species were found in abundance in the locations with Cs_3_Bi_2_Br_9_ and away from g-C_3_N_4_. In other terms, the deposition of Pt took place on Cs_3_Bi_2_Br_9_ more, indicating that the surface electron density was higher in the crystals to induce the photoreduction process, confirming the presence of a Z-scheme heterojunction.

Based on the results collected from various techniques, it was concluded that the construction of a Cs_3_Bi_2_Br_9_/g-C_3_N_4_ composite was beneficial for boosting the photocatalytic CO_2_ reduction activity. Photocatalytic and photoelectrochemical testing demonstrated an enhancement in CO_2_ reduction efficiency, which was explained by an improvement in charge transfer between the two components of the composite. Quenching of PL emission and shifts in XPS binding energies have highlighted a positive change in charge transfer. In addition, energetics analysis of the two components, postreaction stability testing, and photoreduction of Pt nanoparticles prove the preferential movement of electrons toward the Cs_3_Bi_2_Br_9_. These findings, put together, strongly link the improvement in activity to a direct Z-scheme mode of enhanced charge separation. In this mode, photogenerated electrons (e^–^) in g-C_3_N_4_ recombine with the photogenerated holes (h^+^) of the Cs_3_Bi_2_Br_9_. In turn, the remaining photogenerated charges on the surface of the semiconductors are available to participate in CO_2_ reduction (on the Cs_3_Bi_2_Br_9_) and H_2_O oxidation (on the g-C_3_N_4_) such that
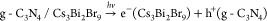
1The photogenerated holes, h+, oxidize water to produce oxygen and H^+^ ions,^7,44^
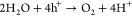
2The generated H^+^ ions along with the photogenerated electrons, e^–^, reduce the CO_2_ to produce CO,^7^
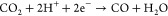
3

## Conclusion

In this study, we successfully demonstrated the synthesis of hybrid organic–inorganic composite heterostructures between layered g-C_3_N_4_ and crystalline Cs_3_Bi_2_Br_9_ at different ratios. Our results showed the highest CO production with 40 wt % Cs_3_Bi_2_Br_9_ crystallized on the g-C_3_N_4_ surface, with a rate of 14.22 (±1.24) μmol CO g^–1^ h^–1^. The improvement in CO production was linked to improved morphologies and charge separation in the composites of g-C_3_N_4_ and Cs_3_Bi_2_Br_9_, following a direct Z-scheme pathway where photoinduced electrons accumulate on Cs_3_Bi_2_Br_9_ and holes on g-C_3_N_4_. This pathway was confirmed with multiple techniques. For example, the constructed energy band diagram using valence band XPS and UV–vis spectroscopy confirmed staggered valence and conduction bands and a deeper Fermi level in g-C_3_N_4_, which would result in a Z-scheme heterojunction upon Fermi level alignment. X-ray photoelectron spectroscopy showed downward band bending in g-C_3_N_4_ and reduction of Bi^3+^ species in Cs_3_Bi_2_Br_9_ to Bi^0^ upon the accumulation of electrons. Moreover, photocatalytic deposition of Pt nanoparticles took place in abundance on the Cs_3_Bi_2_Br_9_ crystals further validating a higher density of electrons on its surface compared to g-C_3_N_4_. Other techniques, such as electron paramagnetic resonance, confirmed the superior properties of the composites. These findings demonstrate the benefits of compositing Cs_3_Bi_2_Br_9_ and g-C_3_N_4_ and open new avenues for their exploitation in CO_2_ conversion.

## Data Availability

The data that support the findings of this study are openly available in a research data repository at https://doi.org/10.6084/m9.figshare.24305635.

## Supporting Information Available

Apparent quantum efficiency calculations; reaction condition optimization results; XRD deconvolution data; steady-state and time-resolved photoluminescence spectra; XPS survey scans and C 1s, N 1s, Cs 3d, Bi 4f, and Br 3d of the pure semiconductors and their composites (not normalized); CHN analysis; summary of XPS peaks; FT-IR and Raman spectroscopy data; mass spectra of ^13^C labeled product; concentration of CO produced from pure g-C_3_N_4_, pure Cs_3_Bi_2_Br_9_, and 40 wt % Cs_3_Bi_2_Br_9_/g-C_3_N_4_ under continuous flow of CO_2_ over 15 h of illumination; XPS spectra before and after stability tests; images of the quartz filter with the pure g-C_3_N_4_, Cs_3_Bi_2_Br_9_, and 40 wt % Cs_3_Bi_2_Br_9_/g-C_3_N_4_ before the reaction and after 1 h of illumination; and normalized XPS scans of Bi 4f peaks (PDF)

## Abbreviations

FTO: fluorine-doped tin oxide
TBAPF_6_: tetrabutylammonium hexafluorophosphate
XRD: X-ray diffraction
DRS: diffuse reflectance spectroscopy
XPS: X-ray photoelectron spectrophotometry
SEM: scanning electron microscopy
TEM: transmission electron microscopy
PL: photoluminescence
FT-IR: Fourier transform infrared spectroscopy
ATR: attenuated total reflection
EDX: energy dispersive X-ray
EPR: electron paramagnetic resonance
PC: photocatalyst
BID: barrier ionization discharge
MS: mass spectrometer
PEC: photoelectrochemical
AQE: apparent quantum efficiency
CB: conduction band
VB: valence band
