# Supplementary material for: Cs3Bi2Br9/g-C3N4 Direct Z-Scheme Heterojunction for Enhanced Photocatalytic Reduction of CO2 to CO
Source: Chem Mater. 2023 Oct 16;35(20):8607–20. doi: 10.1021/acs.chemmater.3c01635 (PMC10601477; doi:10.1021/acs.chemmater.3c01635)
Supplement: Supplementary file 1 — cm3c01635_si_001.pdf [file cm3c01635_si_001.pdf]

Supporting Information

$Cs_3Bi_2Br_9$/g-C_3N_4 direct Z-scheme heterojunction for enhanced photocatalytic reduction of CO_2 to CO

Yasmine Baghdadi, [a] *Filipp Temerov,* [a, b] *Junyi Cui,* [a] *Matyas Daboczi,* [a] *Eduardo Rattner,* [a] *Michael Segundo Sena,* [a, c] *Ioanna Itskou,* [d] *and Salvador Eslava* *[a]

[a] Department of Chemical Engineering and Centre for Processable Electronics, Imperial College London, London SW7 2AZ, United Kingdom. E-mail: s.eslava@imperial.ac.uk

[b] Nano and molecular system (NANOMO) research unit, University of Oulu, Oulu, 90570, Finland

[c] Department of Graduation in Chemical Engineering, Universidade Federal do Rio Grande do Norte/UFRN, 59.078-970 Rio Grande do Norte, Brazil

[d] Barrer Centre, Department of Chemical Engineering, Imperial College London, London SW7 2AZ, United

Apparent quantum yield calculations

To measure the apparent quantum yield (AQE), the sample was tested in the same experimental setup using the same conditions while replacing the 300 W Xe lamp with a 365 nm monochromatic LED lamp. To perform the calculations, the following parameters were used:

Irradiance,	$\boldsymbol{I}$	= 100 mW cm^{-2} = 1000 W m^{-2}
Area of effective light irradiation,	$\boldsymbol{A}$	= $8x10^{-4}$ m^2
Plank's constant,	$\mathbf{h}$	= $6.626x10^{-34}$ J s
Speed of light,	$\mathbf{c}$	= $3x10^{17}$ nm s^{-1}
Wavelength of incident light,	$\boldsymbol{\lambda}$	= 365 nm
Avogadro's number,	$\mathbf{N_A}$	= $6.022x10^{23}$ mol^{-1}

Incident light intensity, $(\boldsymbol{I_0})$ = $\boldsymbol{I}$x$\boldsymbol{A}$

Photon energy, $(\mathbf{E_p})$ = $\frac{\mathbf{hc}}{\lambda} = \frac{(6.626x10^{-34}\ \mathrm{J\ s})x(3x10^{17}\ \mathrm{nm\ s^{-1}})}{365\ \mathrm{nm}}$

Therefore, the number of incident photons per unit time (N_P) can be calculated as:

$$\boldsymbol{N_P} = \frac{\boldsymbol{I_0}}{\boldsymbol{E_P}}$$

The number of moles of incident photons per unit time (M_P) can be calculated by dividing N_P by $\mathrm{N_A}$ such that:

$$\boldsymbol{M_P} = \frac{\boldsymbol{N_P}}{\mathbf{N_A}}$$

Finally, AQE is calculated by dividing the moles of reacted electrons by M_P such that:

$$\mathbf{AQE}\ (\%) = \frac{\mathbf{2xCO_{produced}}}{\boldsymbol{M_P}}\mathbf{x100}$$

Supporting data

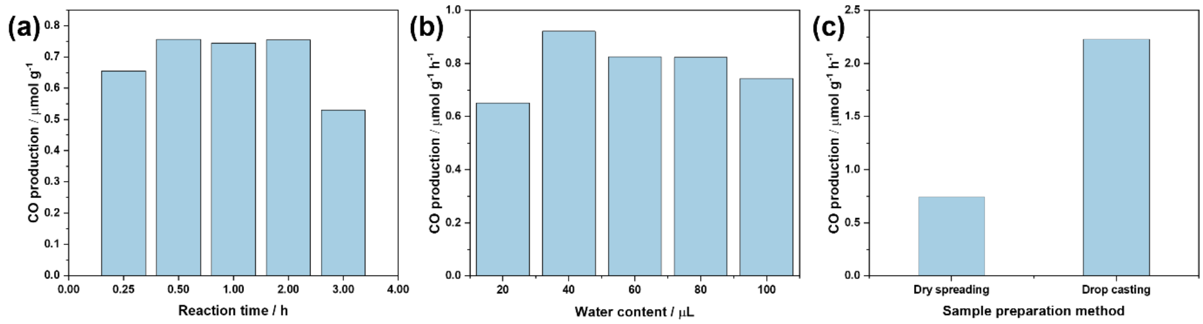

Figure S1: CO production for bulk g-C_3N_4 at different (a) reaction time, (b) water content, and (c) sample preparation methods

For XRD peak analysis, a Gaussian deconvolution was implemented based on the following formula:

$$y = y_0 + \frac{A}{w * \sqrt{\frac{\pi}{4\ln(2)}}} * \exp\left(-4\ln(2) * \frac{(x - x_c)^2}{w^2}\right)$$

where y0 represents the base of the peak, xc is the peak center, A is the area, and w is the full width at half maximum (FWHM)

Table S1: Calculated parameters for the deconvoluted peaks based on the Gaussian model.

Sample		g-C_3N_4 (002) peak				$Cs_3Bi_2Br_9$ (112) peak				$Cs_3Bi_2Br_9$ (201) peak			
		y0	xc	A	w	y0	xc	A	w	y0	xc	A	w
**g-C_3N_4**	**Value**	162.26	27.39	6311.52	1.3								
	**σ**	25.56	0.01	98.34	0.02								
**10 wt%**	**Value**	74.82	27.45	1475.73	0.97	74.82	27.45	387.64	0.18	74.82	27.22	130.13	0.2
	**σ**	11.81	0.01	51.2	0.03	11.81	0.01	33.53	0.01	11.81	0.02	33.08	0.04
**20 wt%**	**Value**	92.7	27.37	1148.64	0.89	92.7	27.37	349.55	0.17	92.7	27.15	115.54	0.18
	**σ**	13.06	0.02	58.38	0.04	13.06	0.01	36.5	0.01	13.06	0.02	34.63	0.05
**40 wt%**	**Value**	90.22	27.47	378.71	0.61	90.22	27.47	445.49	0.16	90.22	27.23	225.55	0.23
	**σ**	14.46	0.08	127.22	0.11	14.46	0.01	64.31	0.01	14.46	0.02	90.06	0.05
**60 wt%**	**Value**	90.08	27.41	322.32	0.46	90.08	27.41	439.95	0.15	90.08	27.17	205.91	0.2
	**σ**	15.45	0.16	298.35	0.15	15.45	0	124.09	0.02	15.45	0.02	187.72	0.07
**80 wt%**	**Value**	73.3	27.39	537.91	0.48	73.3	27.47	352.71	0.15	73.3	27.22	86.36	0.15
	**σ**	13.19	0.03	227.26	0.09	13.19	0.01	132.7	0.02	13.19	0.03	105.45	0.08
**$Cs_3Bi_2Br_9$**	**Value**					84.66	27.44	590.62	0.13	84.66	27.2	507.26	0.13
	**σ**					18.6	0	23.33	0.01	18.6	0	23.1	0.01

*σ represents the standard error

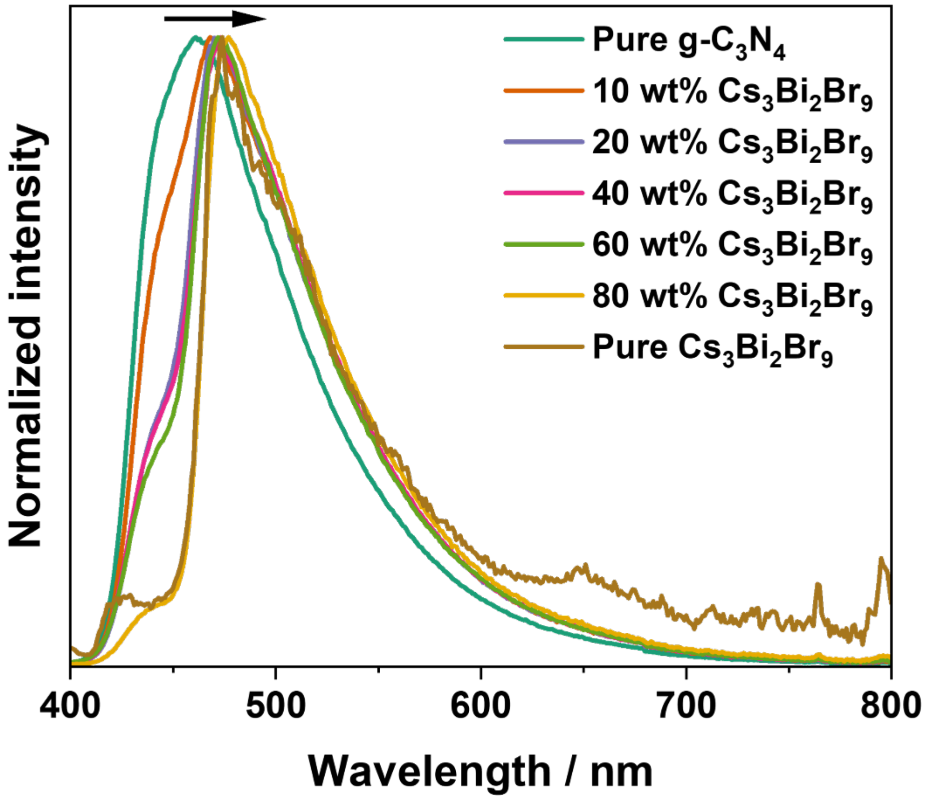

Figure S2: Steady-state PL spectra of the samples normalized to their maximum intensity. Excitation wavelength λ_{ex}= 380 nm laser

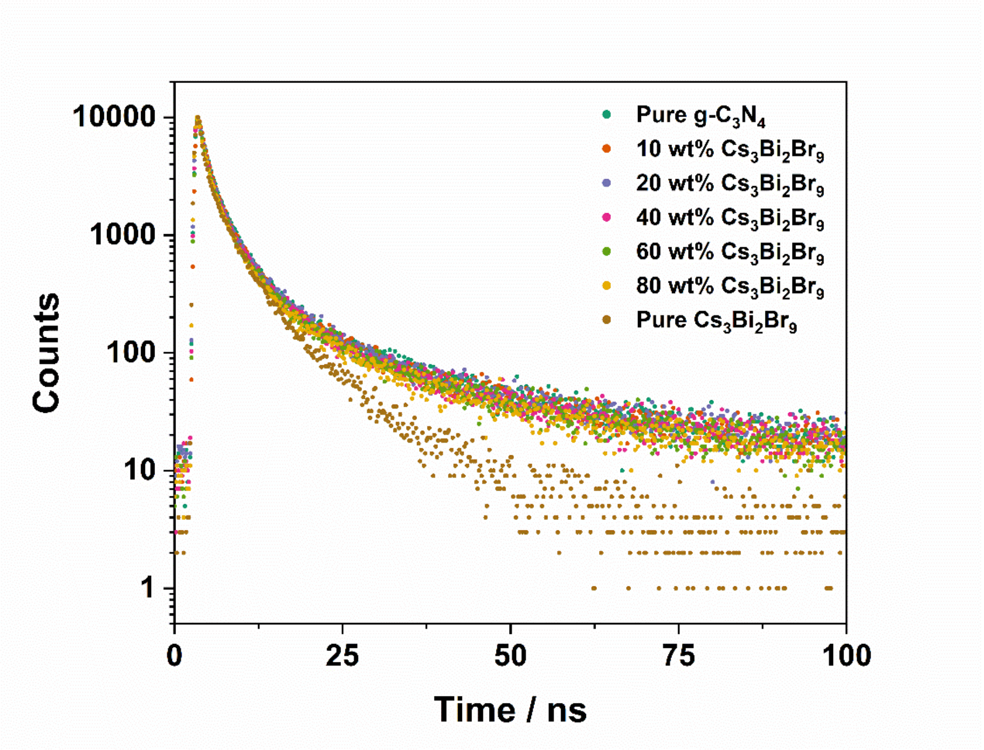

Figure S3: Time-resolved PL spectra. Excitation wavelength λ_{ex}= 405 nm laser.

Table S2: Summary values of TRPL plot fittings of the pure semiconductors and the synthesized composites

Sample	B_1	τ_1 (ns)	B_2	τ_2 (ns)
Pure g-C_3N_4	10422.7	1.913	753.9	13.080
10 wt% $Cs_3Bi_2Br_9$/g-C_3N_4	9702.8	1.866	707.5	12.906
20 wt% $Cs_3Bi_2Br_9$/g-C_3N_4	10098.4	1.799	840.7	11.882
40 wt% $Cs_3Bi_2Br_9$/g-C_3N_4	9802.4	1.822	742.2	12.087
60 wt% $Cs_3Bi_2Br_9$/g-C_3N_4	9138.3	1.727	737.7	11.879
80 wt% $Cs_3Bi_2Br_9$/g-C_3N_4	9934.7	1.735	745.9	11.364
Pure $Cs_3Bi_2Br_9$	9074.1	1.029	2209.3	5.619

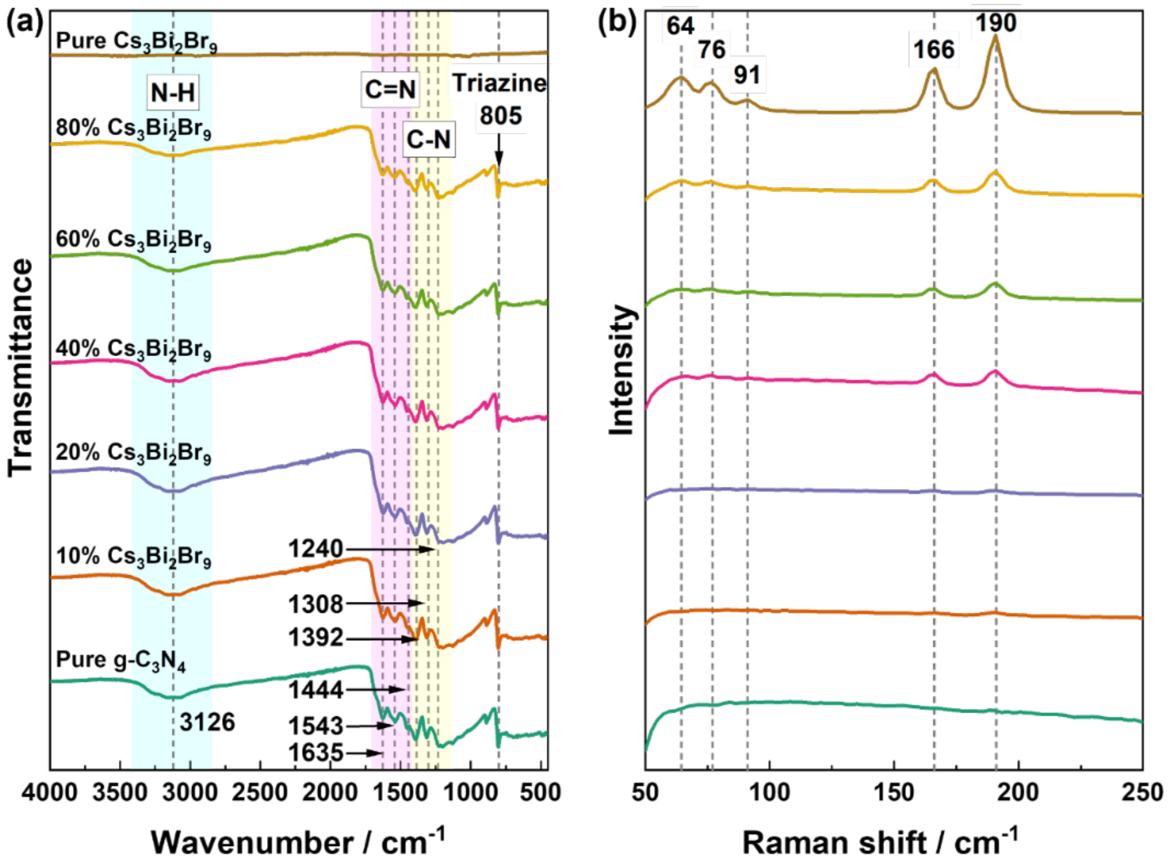

Figure S4: (a) FT-IR and (b) Raman spectra of pure g-C_3N_4, $Cs_3Bi_2Br_9$, and their composites

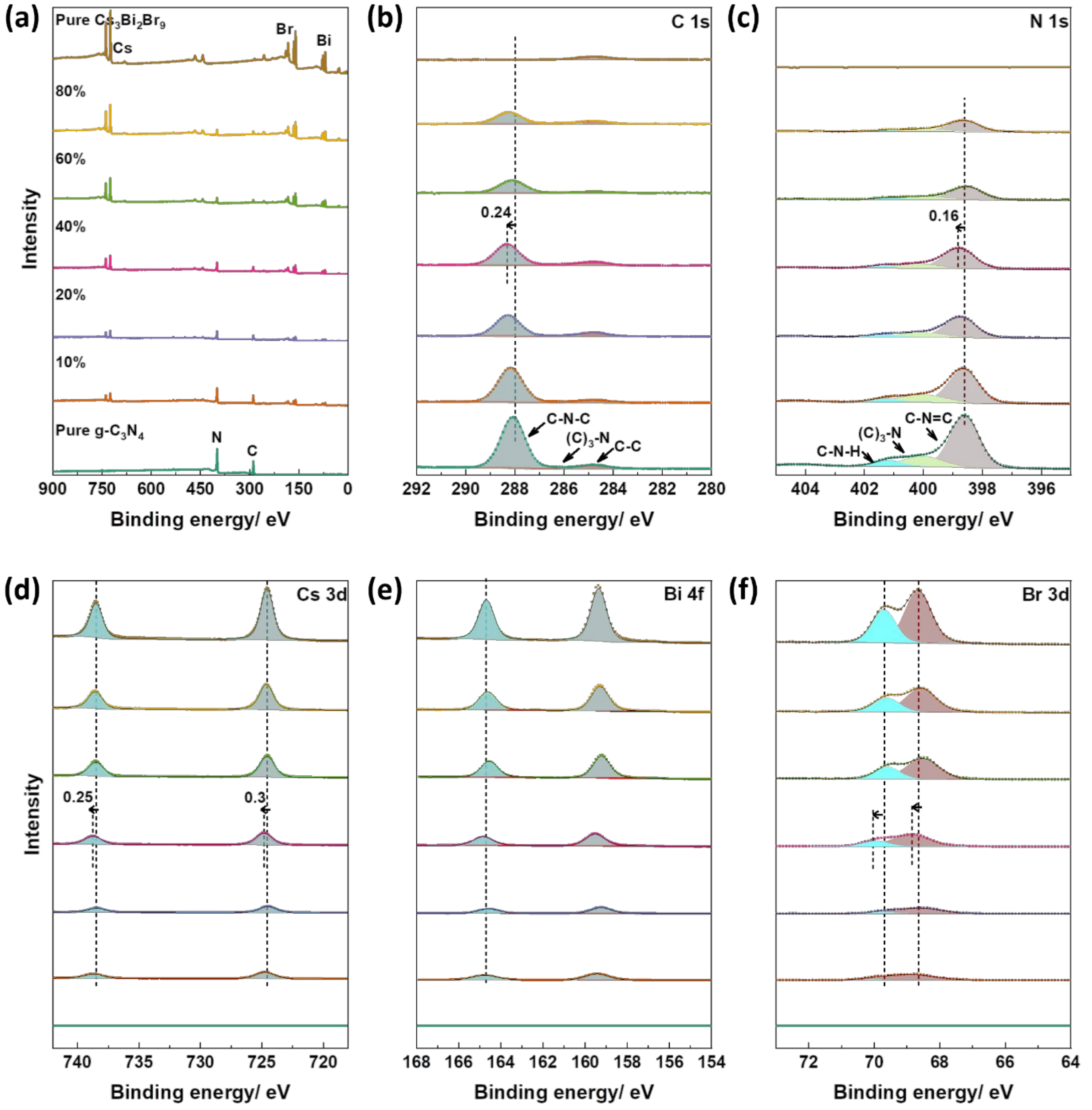

Figure S5: XPS (a) survey scans with scans of (b) C 1s, (c) N 1s, (d) Cs 3d, (e) Bi 4f, and (f) Br 3d of the pure semiconductors and their composites

Table S3: Summary values of expected and found percentages of carbon, nitrogen, and hydrogen in the pure samples as well as the composites using elemental analysis.

Sample	Element	Expected (wt%)	Found (wt%)
Pure g-C_3N_4	C	39.1	34.8
	H	-	1.7
	N	60.9	59.8
10 wt% $Cs_3Bi_2Br_9$/g-C_3N_4	C	35.0	33.0
	H	-	1.6
	N	54.4	55.6
20 wt% $Cs_3Bi_2Br_9$/g-C_3N_4	C	31.2	31.1
	H	-	1.5
	N	48.4	52.1
40 wt% $Cs_3Bi_2Br_9$/g-C_3N_4	C	23.8	22.2
	H	-	1.1
	N	37.1	37.1
60 wt% $Cs_3Bi_2Br_9$/g-C_3N_4	C	15.6	23.4
	H	-	1.2
	N	24.2	39.9
80 wt% $Cs_3Bi_2Br_9$/g-C_3N_4	C	8.0	20.4
	H	-	1.0
	N	12.5	34.6
Pure $Cs_3Bi_2Br_9$	C	0	0.2
	H	0	0.0
	N	0	0.0

Table S4: Summary values of binding energy for the pure semiconductors and the composites

		Peak binding energy (eV)						
		g-C_3N_4	10 wt%	20 wt%	40 wt%	60 wt%	80 wt%	$Cs_3Bi_2Br_9$
C 1*s*	C-C	284.80	284.80	284.80	284.80	284.80	284.80	284.80
	$(C)_3$-N	286.37	286.36	286.45	286.36	286.39	285.49	-
	C-N-C	288.07	288.17	288.26	288.28	288.10	288.29	-
N 1*s*	C-N=C	398.59	398.63	398.68	398.76	398.51	398.69	-
	$(C)_3$-N	400.07	400.03	401.17	401.23	399.94	401.05	-
	C-N-H	401.18	401.24	399.41	400.09	401.16	399.28	-
Cs	3*d*5	-	724.73	724.47	724.78	724.56	724.74	724.62
	3*d*3	-	738.66	738.40	738.70	738.48	738.66	738.54
Bi^{3+}	4*f*7	-	159.42	159.22	159.49	159.21	159.40	159.31
	4*f*5	-	164.75	164.57	164.82	164.54	164.73	164.63
Bi^0	4*f*7	-	157.93	157.98	158.34	158.20	158.23	158.75
	4*f*5	-	163.04	163.09	163.45	163.31	163.34	164.27
Br	3*d*5/2	-	68.93	68.58	68.85	68.50	68.71	68.56
	3*d*3/2	-	70.02	69.70	69.95	69.58	69.79	69.62

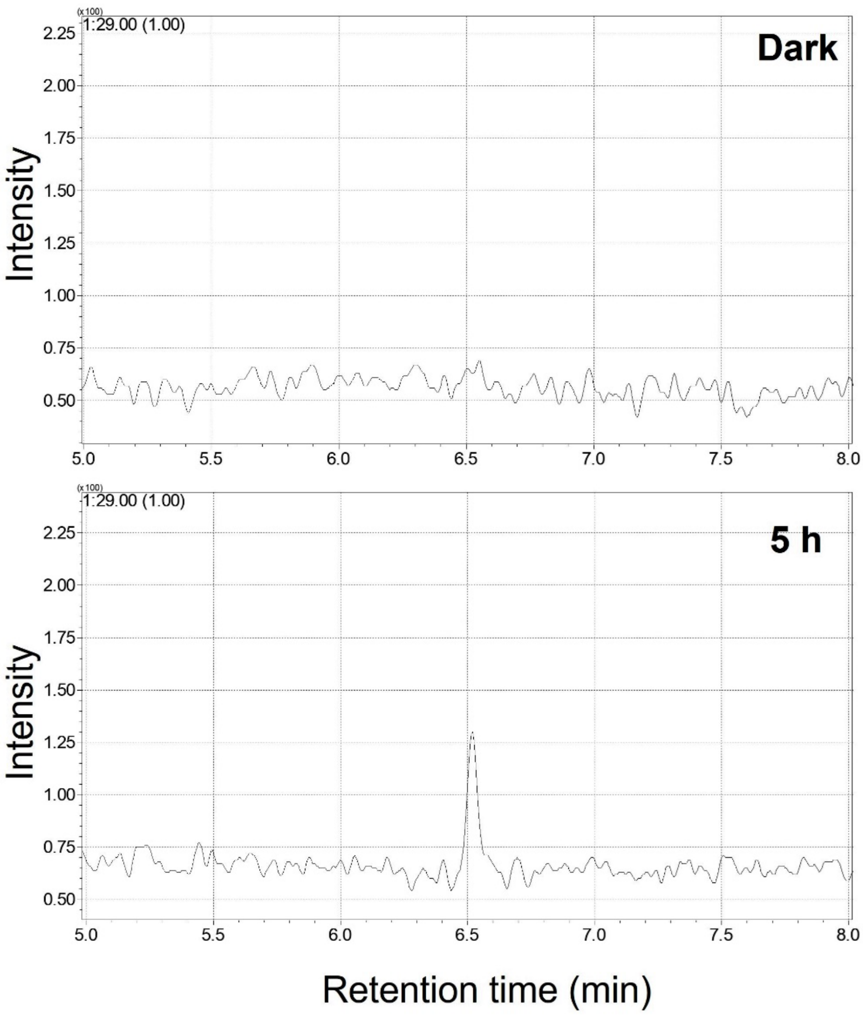

Figure S6: Mass spectroscopy spectrum obtained after performing a reaction with $^{13}CO_2$ after 5 h for illumination at 1 bar for 40 wt% $Cs_3Bi_2Br_9$/g-C_3N_4.

Table S5: Summary of similar photocatalyst composites found in literature.

Photocatalyst	Production (μmol $g^{-1}h^{-1}$)		Medium	Reaction conditions	Light source	Ref. in manuscript
$Cs_3Bi_2Br_9$/g-C_3N_4	CO	14.22	CO_2 (g) saturated with H_2O	ambient conditions	300 W Xe, AM 1.5G, 100 mW cm^{-2}	This work
$Cs_2AgBiBr_6$-gC_3N_4	CO	12.14	Isopropanol	25 ℃	250 W Hg (285≤ λ ≤ 700 nm)	[11]
	CH_4	8.85				
MCM-41-$Cs_3Bi_2Br_9$	CO	17.24	CO_2 (g) saturated with H_2O	20 ℃	300 W Xe ($\lambda \geq$ 420 nm) , 350 mW cm^{-2}	[9]
$Cs_3Bi_2Br_9$/$BiVO_4$	CO	28.13	CO_2 (g) saturated with H_2O	ambient conditions	300 W Xe, AM 1.5G, 100 mW cm^{-2}	[12]
$Cs_3Bi_2Br_9$/Bi_2WO_6	CO	220.1	CO_2 (g), $NaHCO_3$, and H_2SO_4	20 ℃	300 W Xe, AM 1.5G, 100 mW cm^{-2}	[13]
CeO_2/g-C_3N_4	CO	0.590	CO_2 (g) saturated with H_2O	25 ℃	300 W Xe	[14]
	CH_4	0.694				
TiO_2 modified g-C_3N_4	CO	56.2	NaOH and triethanolamine	0.4 MPa	8 W UV	[15]
	CH_4	72.2				
α-Fe_2O_3/g-C_3N_4	CO	27.2	$NaHCO_3$, and H_2SO_4	1 bar and 20 ℃	Xe, 0.21 W cm^{-2}	[8]
$CoNiS_x$/g-C_3N_4	CO	11.77	H_2SO_4/H_2O mix (1:1) with $NaHCO_3$	-	300 W Xe, 850 mW cm^{-2}	[16]
	CH_4	0.904				
$CsPbBr_3$/g-C_3N_4	CO	28.5	CO_2 (g) saturated with H_2O	-	300 W Xe ($\lambda \geq$ 420 nm)	[17]
$LaCoO_3$/g-C_3N_4	CO	135.2	CO_2 (g) saturated with H_2O	0.3 bar	35 W Xe, 20 mW cm^{-2}	[18]

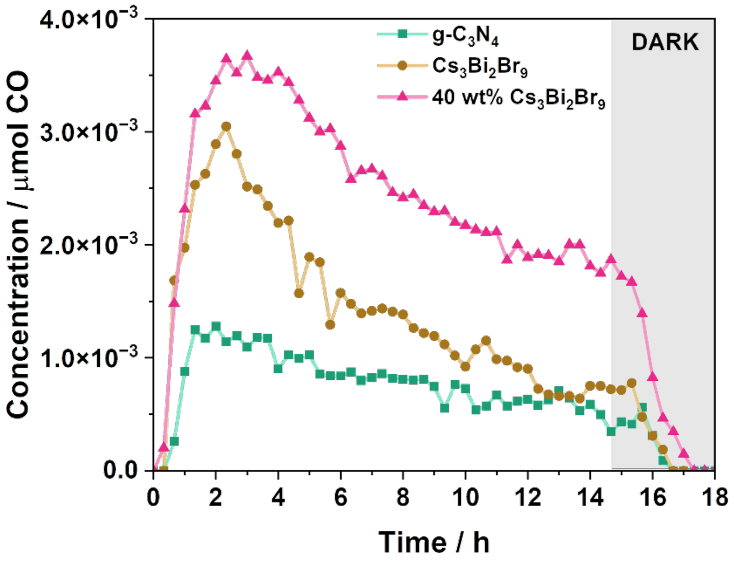

Figure S7: Concentration of CO produced from pure g-C_3N_4, pure $Cs_3Bi_2Br_9$, and 40 wt% $Cs_3Bi_2Br_9$/g-C_3N_4 under continuous flow of CO_2 over 15 h of illumination.

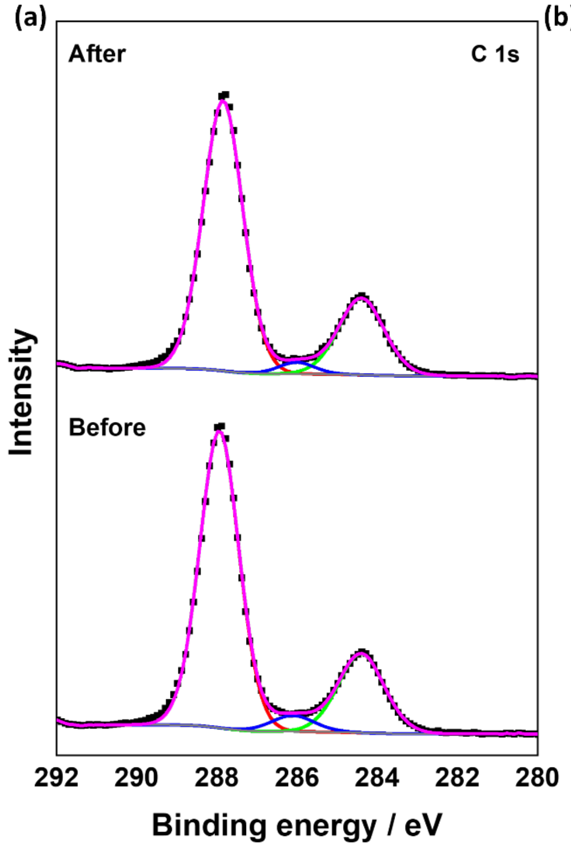

Figure S8: XPS (a) C 1s and (b) N 1s of the pure g-C_3N_4 before and after the 1 h reaction

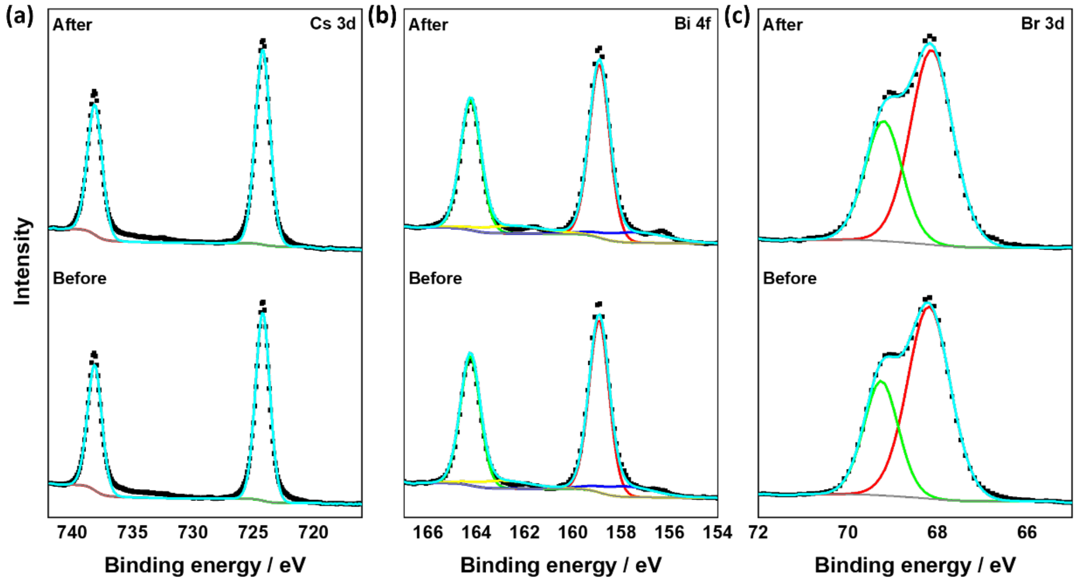

Figure S9: XPS (a) Cs 3d, (b) Bi 4f and (c) Br 3d of the pure $Cs_3Bi_2Br_9$ before and after the 1 h reaction

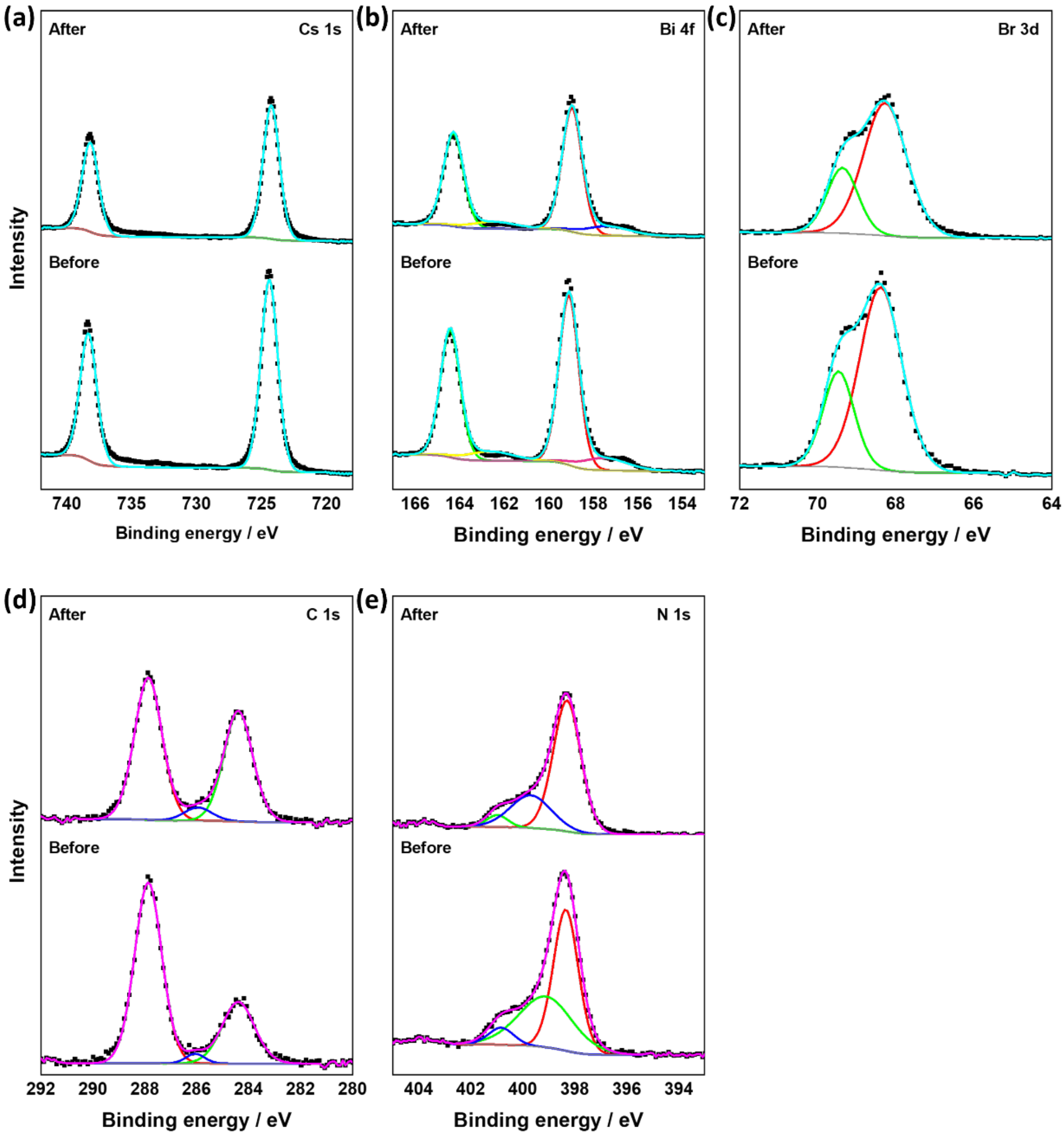

Figure S10: XPS (a) Cs 3d, (b) Bi 4f, (c) Br 3d, (d) C 1s and (e) N 1s of the 40 wt% $Cs_3Bi_2Br_9$/g-C_3N_4 before and after the 1 h reaction

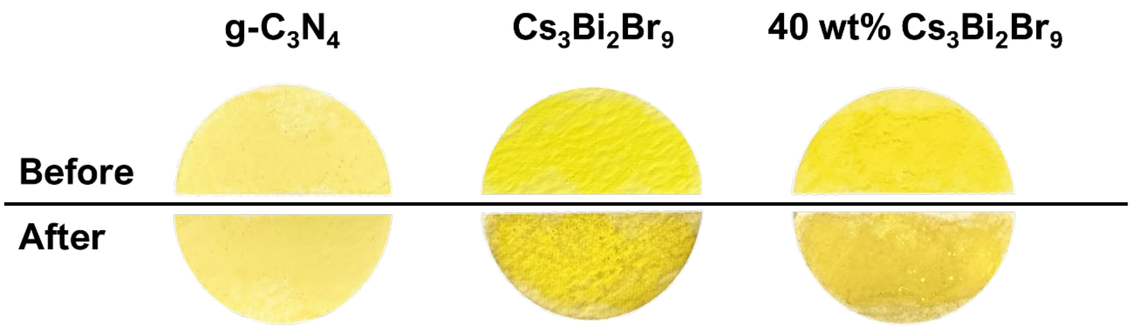

Figure S11: Images of the quartz filter with the pure g-C_3N_4, $Cs_3Bi_2Br_9$, and 40 wt% $Cs_3Bi_2Br_9$/g-C_3N_4 before the reaction and after 1 h of illumination

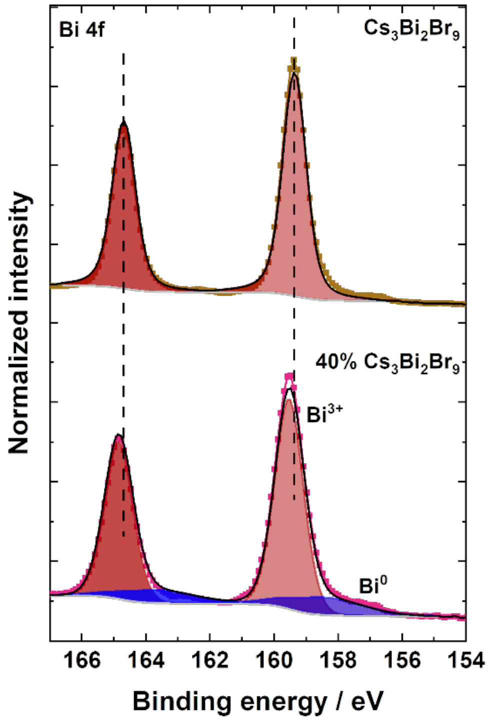

Figure S12: Normalized XPS scans of Bi 4f peaks of pure $Cs_3Bi_2Br_9$ and 40 wt% $Cs_3Bi_2Br_9$ composite
